# **Catalytic degradation of norfloxacin using persulfate activation by Ni-Fe layered double hydroxide catalyst supported on activated carbon**

**DOI:** 10.1038/s41598-025-89106-w

**Published:** 2025-02-11

**Authors:** Adel Adly, Mona M Galal, Minerva E Matta

**Affiliations:** Sanitary and Environmental Engineering Division, Faculty of Engineering, Cairo University, Giza, 12613 Egypt

**Keywords:** Advanced oxidation process, Norfloxacin degradation, Persulfate activation, Sulfate radicals, Wastewater treatment, NiFe-LDH@AC composite, Biochemistry, Catalysis, Chemical biology, Chemical engineering

## Abstract

This study investigated the catalytic degradation of Norfloxacin (NOR), a persistent fluoroquinolone antibiotic, using a novel Ni-Fe Layered Double Hydroxide supported on Activated Carbon (NiFe-LDH@AC) as a catalyst. The composite was designed to activate persulfate (PDS) and generate sulfate radicals for NOR degradation in aqueous solutions. Characterization techniques such as XRD, SEM, EDS, TEM, FTIR, and BET confirmed the successful synthesis and structural integrity of the composite. The optimal degradation was achieved with a NiFe-LDH@AC ratio of 2:1, 0.3 g/L catalyst dosage, and 1 g/L PDS, resulting in 86% NOR removal efficiency within 60 min at neutral pH and ambient temperature for an initial concentration of 50 mg/L, and 100% removal for initial concentrations of 10 mg/L and 20 mg/L under the same conditions. The activation energy of the reaction was calculated as 58.27 kJ/mol. Radical scavenging experiments identified sulfate (SO₄˙⁻) and hydroxyl (^•^OH) radicals as the dominant reactive species, but the SO₄˙⁻ played a larger role. Furthermore, the catalyst exhibited good reusability, maintaining 75% degradation efficiency after four cycles, and showed minimal metal leaching. The study also proposed a mechanism for PDS activation using XPS analysis and suggested NOR degradation pathways through LC-ESI-MS/MS analysis. Moreover, the NiFe-LDH@AC/PDS system demonstrated 84% NOR degradation and 55% COD removal in real treated wastewater. Results demonstrated that the NiFe-LDH@AC composite effectively activated PDS, achieving high NOR removal efficiency, making it a promising sustainable material for wastewater treatment.

**Supplementary Information:**

The online version contains supplementary material available at 10.1038/s41598-025-89106-w.

## Introduction

Antibiotics are vital for human, animal, agricultural, and aquaculture health, but their global consumption has surged from 21.1 billion DDDs in 2000 to 34.8 billion by 2015^1,2^. With extended half-lives of up to 77 days^3^, their widespread use and production contribute to antibiotic-resistant bacteria, posing serious risks to aquatic ecosystems^4^. Traditional wastewater treatment methods struggle to effectively remove these persistent compounds^5^.

Norfloxacin (NOR), a fluoroquinolone antibiotic, is widely used for bacterial infections but tends to persist and accumulate in the environment more than other antibiotics^6^. Approximately 75% of NOR is not metabolized in the human body and is released into aquatic environments^1^. Consequently, NOR is commonly detected in wastewater treatment plants, medical wastewater, and surface water, posing significant risks of antimicrobial resistance and environmental harm, which threaten both ecosystems and human health^1,3^.

Various techniques have been developed to remove antibiotics from wastewater, but each has limitations. Physical methods, such as adsorption, flocculation, and membrane separation, are easy to implement but merely transfer pollutants from the liquid phase to the solid phase, causing potential secondary contamination^7–10^. Biological methods are widely used but fail to eliminate recalcitrant pollutants like pharmaceuticals due to their low biodegradability^3,11^. Chemical methods are more effective for removing persistent pollutants^3^, highlighting the need for advanced, efficient treatment approaches to address environmental challenges.

Recently, Advanced Oxidation Processes (AOPs) have emerged as effective, environmentally friendly, and cost-efficient methods for decomposing refractory pollutants^10^. AOPs encompass a range of technologies, including photocatalysis, Fenton reactions, sulfate radical oxidations, electrochemical oxidation, ultrasound irradiation, and ozonation, which have demonstrated significant success in wastewater treatment^12^. These processes generate highly reactive free radicals that act as strong oxidants, enabling the effective degradation of persistent organic pollutants in water^6^.

Recent advancements in AOPs have shown great potential for the efficient degradation of norfloxacin (NOR) and other antibiotics. For instance, Diao et al.^13^achieved 99% amoxicillin degradation using a TiO₂@nZVI/PDS system under UV irradiation through photocatalysis and sulfate radical oxidation. Similarly, PMS-assisted photocatalysis with a calcium-based Ag₃PO₄ composite under visible light achieved nearly 99% NOR removal through reactive species like SO₄˙⁻ and HO˙^14^​. These studies underscore the importance of tailored catalysts and oxidant activation strategies for optimizing AOPs in practical applications.

Heterogeneous Fenton-like technology has gained attention for its versatility in degrading various contaminants, high efficiency, and catalyst reusability^15^. This system relies on an oxidant and a catalyst, with common oxidants such as hydrogen peroxide (•OH), peroxymonosulfate (PMS), and peroxydisulfate/persulfate (PDS/PS)^12^. These oxidants generate reactive oxygen species (ROS), including hydroxyl radicals (•OH) and sulfate radicals (SO_4_^•−^), which exhibit strong oxidative capabilities to break pollutants into less harmful products like inorganic salts, CO₂, and H₂O^16,17^.

Sulfate radicals (SO_4_^•−^) are particularly advantageous due to their higher redox potential (E₀ = 2.60 − 3.10 eV), longer lifetime (t₁/₂ = 30 − 40 µs), greater selectivity, and broader operative pH range (pH 2.0–8.0)^17,18^. These radicals are generated by activating PDS or PMS using various techniques, such as transition metals, chemical activators, external energy sources (UV, ultrasound, thermal), and carbon-based materials like graphene oxide and biochar^17^.

Transition metal-based heterogeneous catalysts are widely used in Fenton-like systems due to their efficiency, cost-effectiveness, and recyclability^17,19^. Common transition metals, such as cobalt, iron, copper, manganese, and nickel, activate oxidants via redox cycles between lower (M^n+^) and higher valence states (M^(*n*+1)+^)^3^. However, monometallic catalysts often face challenges such as diminished activity due to inefficient valence state conversion and toxic metal leaching from unstable crystalline structures, limiting real-world applicability^15,17^.

Layered double hydroxides (LDHs) address these limitations due to their structural stability, resistance to pH and thermal variations, high anion exchange capacity, and surface area^17,20^. LDHs consist of divalent and trivalent metal hydroxide layers and exchangeable interlayer anions, facilitating redox cycles for effective catalysis^21^. For instance, Gong et al.^22^ reported that CoFe-LDH could effectively activate PMS to degrade 99% of Rhodmine B in 10 min. Guo et al.^23^ found that CoCu-LDH catalyst achieved high degradation of lomefloxacin by activating PMS. Hou et al.^24^ synthesized MnFe-LDHs for activating PMS and degrading acid orange 7 (AO7). The study demonstrated a high degradation efficiency of 97.56% for pollutants, achieved through the generation of •OH/SO_4_^•−^radicals from the redox reaction between Fe/Mn ions and PMS^24^.

Layered double hydroxides (LDHs) face challenges such as agglomeration, which reduces reactivity, and decomposition in acidic conditions, which can release heavy metals^20,21^. To address these issues, carbon materials like activated carbon^19^, graphene oxide^21^, and biochar^18^, are often employed as support materials for metal-based catalysts. Metal-carbon composites demonstrate a synergistic effect that enhances the redox cycle, minimizes metal leaching, improves stability, and promotes the production of sulfate radicals (SO_4_^•−^) in the PDS activation process^17,25^. For instance, CoFe-LDH supported on biochar in combination with PMS achieved 100% Dimethyl Phthalate degradation compared to 62% for CoFe-LDH alone^18^. Similarly, the incorporation of carbon black into ZnFe-LDH enhanced ibuprofen degradation efficiency to 75.4%, compared to 60.6% for the carbon-free compound after 90 min of stirring^26^.

This study presents NiFe-LDH@AC, a novel composite material developed through a co-precipitation method that combines the redox-active properties of NiFe-LDH with the high surface area and stability of activated carbon (AC). This synergy enables efficient heterogeneous catalysis for activating PDS and generating reactive sulfate radicals to degrade the persistent antibiotic norfloxacin. Advanced characterization techniques revealed the composite’s structure-activity relationship. The effects of various reaction parameters, including catalyst dosage, PDS concentration, pH, NOR concentration, and temperature, were systematically optimized. The composite’s stability and reusability were also evaluated, addressing traditional catalysts’ limitations. A detailed mechanism of PDS activation and NOR degradation pathway was proposed based on LC-ESI-MS/MS analysis, demonstrating the composite’s ability to break down complex pollutants into less harmful products. This work represents a significant advancement in the field of advanced oxidation processes, offering a robust and practical solution for addressing the challenges of removing recalcitrant pollutants from aqueous environments.

## Materials and methods

### Chemicals

The reagents employed in the experimental work were of analytical grade, ensuring their high purity and quality. Iron (III) chloride hexahydrate (FeCl_3_·6H_2_O, 98.5%), Nickel (II) nitrate hexahydrate (Ni(NO_3_)_2_·6H_2_O, 98.5%), and Potassium peroxydisulfate (PDS) (K_2_S_2_O_8_, 99.5%) were obtained from LobaChemie Ltd, (Mumbai, India). Ethyl Alcohol Absolute (Ethanol) (C_2_H_5_OH, 99.5%), and Tertiary-butylalcohol (TBA) (C(CH_3_)_3_OH, 99.0%) were purchased from El Nasr pharmaceutical chemical Co., (Cairo, Egypt). Methanol (CH_3_OH, 99.5%), Sodium hydroxide (NaOH), and Hydrochloric acid (HCl) were obtained from Techno Pharmchem, (New Delhi, India). Activated carbon (AC) was purchased from Advent chembio., (Mumbai, India). Finally, Norfloxacin Antibiotic (NOR, > 98%) was purchased from Solarbio Science and Technology Co. Ltd (Beijing, China). Deionized (DI) water was utilized in all experiments.

### Preparation of NiFe-LDH@AC

The NiFe-LDH@AC catalyst was prepared using co-precipitation method with varying mass ratios of AC to NiFe-LDH (1:2, 1:1, 2:1)^19,25^. Firstly, FeCl_3_·6H_2_O (3 mmol) and Ni(NO_3_)_2_·6H_2_O (6 mmol) were dissolved in 60 mL DI water and stirred at room temperature to create a homogeneous mixture (Solution A). Simultaneously, a solution of NaOH (25 mmol) in 60 mL of DI water was prepared (Solution B). AC was added to a beaker containing 60 mL of DI water and stirred for 30 min (Solution C). Secondly, Solution A and Solution B were added dropwise to Solution C while stirring with a magnetic stirrer of speed 150 rpm. The pH of the slurry was adjusted to around 10 by controlling the rate of droplet addition. This step facilitated the synthesis of LDH and its attachment to the surface of AC. Thirdly, the suspension was aged at 60 °C for 12 h. The resulting precipitate was separated by filtration, washed with ultrapure water and ethanol until the pH of the supernatant reached neutrality, and then dried at 60 °C in an oven for 24 h. The final product obtained from this process was called NiFe-LDH@AC^19^.

NiFe-LDH composites without AC was also prepared. The same synthesis procedures were repeated without adding Solution C in the 500 mL beaker.

### Characterization of catalyst

The different samples (AC, NiFe-LDH, NiFe-LDH@AC) were characterized by X-ray diffraction (XRD D8 ADVANCE, BRUKER) to determine the crystal pattern using Cu Kα radiation (λ = 0.15418 nm) with 2θ range from 10° to 90°. Scanning electron microscope (SEM, Quanta FEG 250) was used to investigate the morphology and structure of the different samples, while EDX spectrum was used for NiFe-LDH@AC composite. Transmission electron microscopy (HRTEM, JEM-2100) was employed for NiFe-LDH@AC composite for more insight into the detailed structures and to show the characteristic spacing of lattice planes of NiFe-LDH@AC. The infrared spectrum of the samples was recorded on a Fourier-transform infrared spectroscopy (FT/IR-4600, JASCO) to further identify the molecular structure and functional groups of the samples. Brunaure-Emmett-Teller (BET) was measured with Quantachrome TouchWin (Ver. 1.21) from N_2_ adsorption-desorption isotherms at 77 K to determine the specific surface area, pore size, and pore volume of the samples. The NiFe-LDH@AC catalyst with mass ratio (2:1) was found to be the optimum composition, so that it was used in the composite characterization.

### Experimental procedure and analysis

The degradation experiments of NOR using the synthesized NiFe-LDH@AC composite were conducted in 250 mL conical flasks. The flasks were placed in a rotary shaker at 160 rpm and room temperature for a duration of 60 min. Initially, an experiment was conducted to determine the optimal mass ratio for the LDH@AC composite. The adsorption properties of different samples (AC, NiFe-LDH, and NiFe-LDH@AC) were evaluated through batch runs. Additionally, the catalytic degradation performances of these samples were examined by introducing PDS during the batch experiments.

In detail, a 100 mL solution of NOR (50 mg/L) was added to the reactor along with a specific amount of catalyst (0.1 g/L) and PDS (1 g/L) at an initial pH of 7, marking the start of the reaction. Liquid samples (5 mL) were collected at regular intervals (5, 15, 30, and 60 min), filtered through a 0.22 μm membrane filter to remove solids, and rapidly mixed with 2.5 mL of methanol to quench the reaction. Subsequently, the samples were analyzed using a high-performance liquid chromatograph (Agilent 1260 Infinity II Prime LC) at a wavelength of 272 nm to measure the remaining concentration of NOR. The batch experiments explored the effect of various reaction parameters on NOR degradation, including catalyst dose, PDS concentration, initial pH value, NOR initial concentration, and reaction temperature. The reaction temperature was controlled using DAIHAN Scientific water bath. The degradation kinetics of NOR was studied by fitting the data to pseudo-first-order model and the reaction rate constants were estimated according to Eq. 1^21^. Moreover, the activation energy was evaluated by Arrhenius equation for activating the PDS using LDH catalyst Eq. 2^27^.

$$\:K=\frac{Ln\frac{{C}_{t}}{{C}_{o}}}{t}\dots\:\dots\:\dots\:\dots\:\:\:\dots\:\dots\:\left(1\right)$$

$$\:Ln\:K=Ln\:A-\:\frac{{E}_{a}}{RT}\dots\:\dots\:\left(2\right)$$

where k represents the reaction rate constant; *C*_*0*_ and *C*_*t*_are the initial concentration and concentration of sampling time^21^. A is the frequency factor; E_a_ is the activation energy, which is the minimum energy required for a reaction to occur; R is the ideal gas constant (8.314 J/(mol·K)); T represents the absolute temperature in Kelvin (K).

X-ray photoelectron spectroscopy (Thermo-Scientific K-Alpha XPS) was used to investigate the mechanism of PDS activation by analyzing the change in the surface elemental compositions in fresh and used catalyst samples (NiFe-LDH@AC). The radicals quenching experiments were conducted using the same procedures with the addition of radical scavengers, such as methanol (MeOH), and tertiary-butyl alcohol (TBA) for detecting the dominant reactive oxygen species (ROS) generated in the system.

Moreover, the concentration of leaching heavy metals (Ni and Fe) in the solution at various pH values for both LDH and LDH@AC composites were detected by Agilent 5100 Synchronous Vertical Dual View (SVDV) ICP-OES. For the reusability test, the samples (NiFe-LDH@AC) were collected, rinsed, and dried at 40 ◦C drying oven for four successive runs. Furthermore, the possible degradation pathways and NOR intermediates were studied using liquid chromatography–electrospray ionization–tandem mass spectrometry (LC-ESI-MS/MS) (ExionLC AC and SCIEX Triple Quad 5500 + MS/MS systems). Finally, 100 mL treated wastewater sample (from Zenin WWTP, Giza) with COD = 62 mg/L mixed with 100 mL NOR solution (COD = 90 mg/L) was studied to show the effectivity of the system in the real wastewater.

## Results and discussion

### Characterization

#### X-ray diffraction (XRD)

**Fig. 1:**
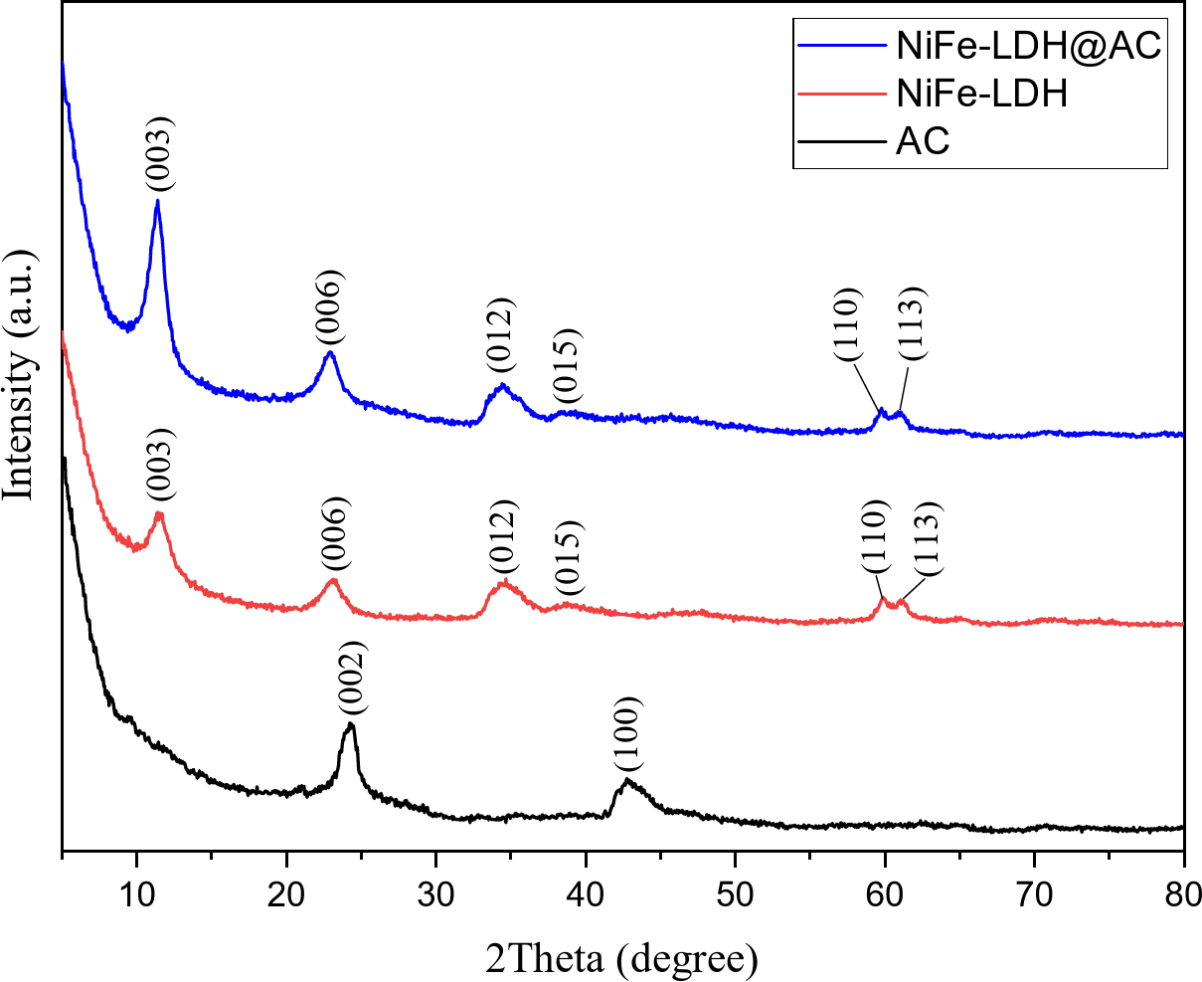
XRD patterns of AC, NiFe-LDH, and NiFe-LDH@AC.

The XRD analysis was employed to assess the crystallinity of the samples, with the results presented in Fig. 1. The diffraction peaks for pure activated carbon (AC) appeared at 24.40° and 42.75°, corresponding to the (002) and (100) planes of graphite carbon, respectively^19^. The observed XRD patterns revealed the characteristic diffraction peaks of the LDH structure with a hexagonal phase^28^. The peaks located at 11.4°, 22.8°, 34.4°, 39.3°, 59.78°, and 60.88° can be indexed to the (003), (006), (012), (015), (110), and (113) planes, respectively, aligning with the classical model of the nickel iron hydrotalcite structure (JCPDS: 40–0215)^29^. The diffraction peaks of LDH@AC and LDH were the same with higher intensity for the composite, which could be attributed to LDH particles better dispersion on the AC surface, resulting in a more uniform structure that gave rise to more intense diffraction peaks^19^. The absence of (002) and (100) diffraction peaks in the NiFe-LDH@AC composite was attributed to the amorphous and porous characteristics of activated carbon (AC), which, in conjunction with the dominance of the highly ordered crystalline LDH structure, suppressed the diffraction signals from these planes^30^.

These findings confirm that the NiFe-LDH was successfully synthesized for peroxydisulfate (PDS) activation, demonstrating a highly ordered and stable composite structure suitable for catalytic applications.

#### Scanning Electron Microscopy - Energy Dispersive Spectroscopy (SEM - EDS)

**Fig. 2:**
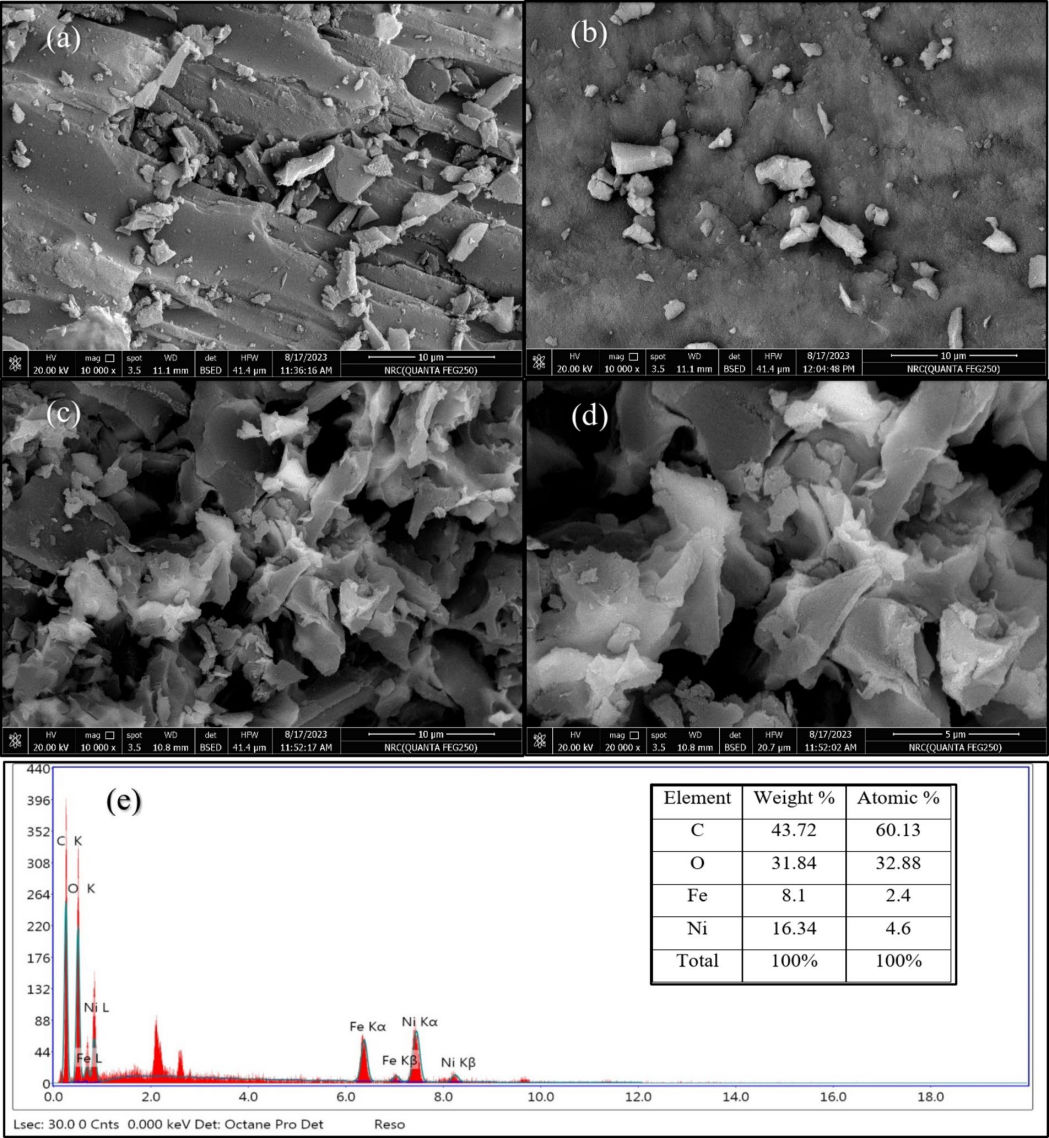
SEM images of (**a**) AC, (**b**) NiFe-LDH, (**c**,** d**) NiFe-LDH@AC, and (**e**) EDS for NiFe-LDH@AC.

The morphology of the synthesized samples was investigated using SEM. The activated carbon (AC), as shown in Fig. 2(a), exhibited a smooth and porous surface, making it an ideal support material for catalysts. The NiFe-LDH particles, Fig. 2(b), displayed a typical layered structure with a plate-like morphology. However, these particles tended to aggregate, forming larger clusters. This aggregation behavior is consistent with the known morphological characteristics of LDHs^18^. When NiFe-LDH was loaded onto the AC support as seen in Fig. 2(c, and d), the SEM images revealed a more even distribution of the LDH particles on the AC matrix, effectively reducing aggregation. This loading process also resulted in a rougher surface for the composite material compared to the pure LDH sample, suggesting an increase in the specific surface area. This enhanced surface area could provide more active sites for catalytic reactions, potentially improving the catalytic performance of the NiFe-LDH@AC composite^19^.

The EDS analysis, as shown in Fig. 2(e), confirmed the presence of carbon (C), oxygen (O), iron (Fe), and nickel (Ni) elements in the NiFe-LDH@AC nanocomposite. The atomic percentages of these elements were determined to be 60.13% for C, 32.88% for O, 2.4% for Fe, and 4.6% for Ni. The high proportions of carbon and oxygen were attributed to the presence of the activated carbon (AC) component. The molar ratio of Ni to Fe was about to be 2.00, which closely matched the ratio used during the synthesis process.

#### High Resolution Transmission Electron Microscopy (HRTEM)

**Fig. 3:**
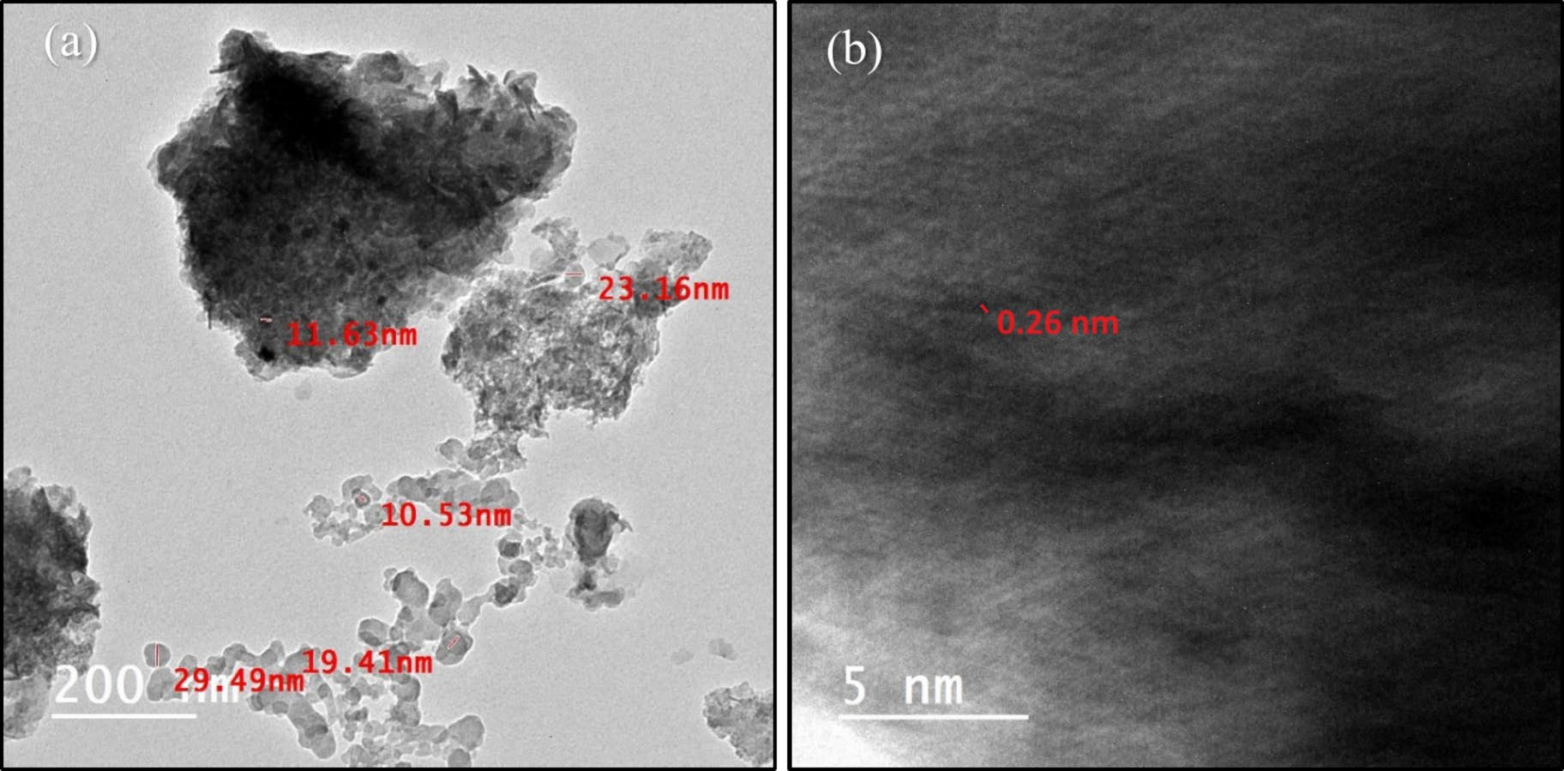
(**a**) TEM image of NiFe-LDH@AC, (**b**) HRTEM image of NiFe-LDH@AC.

TEM analysis was conducted for a deeper understanding of the microstructures of the synthesized NiFe-LDH@AC nanocomposites. Figure 3a revealed that the nanocomposites comprised both sheet-like structures, characteristic of AC, and hexagonal structures, typical of NiFe-LDH. These hexagonal NiFe-LDH plates, with dimensions ranging from 10 to 50 nanometers, were observed to be evenly distributed across the AC surface. The High-Resolution TEM image of the NiFe-LDH@AC nanocomposite, shown in Fig. 3b, displayed a characteristic lattice spacing of 0.26 nm. This spacing aligned well with the (0 1 2) lattice planes of NiFe-LDH, confirming the successful synthesis of the LDH nanocomposite^28^. This observation was consistent with the results obtained from XRD analysis.

#### Fourier-Tansform Infrared Spectroscopy (FTIR)

**Fig. 4:**
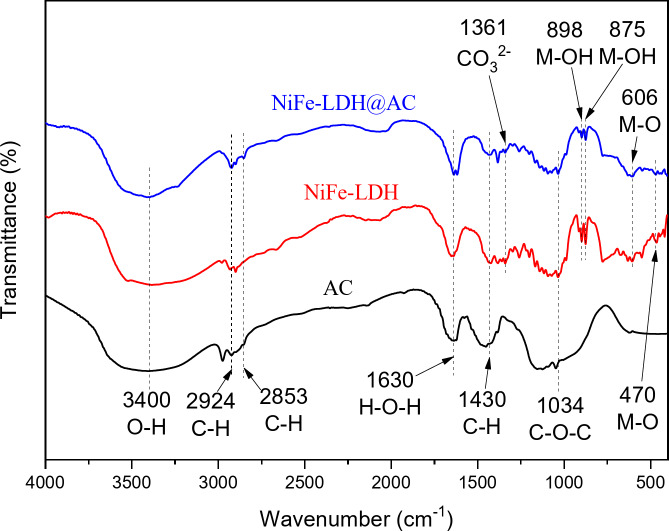
FTIR spectra of AC, NiFe-LDH, and NiFe-LDH@AC composite.

The FTIR spectra of the AC, NiFe-LDH, and NiFe-LDH@AC samples provided a comparative analysis of the chemical bonding and functional groups in each sample. As shown in Fig. 4, a broad peak around 3400 cm⁻¹ indicated O-H stretching vibrations of hydroxyl groups in the LDH structure, as well as adsorbed water molecules^30,31^. The peaks observed at 2924 cm⁻¹, 2853 cm⁻¹, 1430 cm⁻¹, and 1034 cm⁻¹ belonged to the C-H vibrations of CH_2_ and CH_3_groups, along with the C-O-C stretching vibration found in carbon materials, indicating that both AC and NiFe-LDH@AC composites comprised of carbon structures^18,19^. However, a slight shift in the C-O-C stretching band (from 1049 cm⁻¹ to 1034 cm⁻¹) was observed after NiFe-LDH was supported on the AC matrix, indicating that NiFe-LDH was attached to the AC surface through interactions of these functional groups^18^. The peak observed at 1630 cm⁻¹ represented the H-O-H bending vibrations of water molecules, suggesting the presence of adsorbed or interlayer water molecules^29,31^. The peak at around 1360 cm⁻¹ was attributed to carbonate (CO_3_²⁻) ions intercalated within the LDH layers^19,29^. The persistence of the carbonate peak in the composite indicated that the structural integrity of the LDH after incorporation onto AC. The vibrating bands detected in the range of 1000 to 400 cm⁻¹ were attributed to the vibrations of metal–oxygen (M–O) or metal hydroxyl (M–OH) groups (M = Ni or Fe)^31^. Overall, the FTIR analysis confirmed that key structural elements of NiFe-LDH were retained in the NiFe-LDH@AC nanocomposite, with peaks shifts and intensity changes indicating successful integration and interaction between NiFe-LDH and the AC surface.

#### Brunauer-Emmett-Teller (BET)

The N_2_adsorption–desorption isotherms for the different samples were shown in Fig. S2. The isotherms, for the three samples, generally displayed a type IV behavior, suggesting the presence of mesopores^18^. The texture characteristics of the three samples, as determined by the BET method, are presented in Table 1. The LDH sample had a much lower surface area and pore volume compared to AC, reflecting its layered structure and less porosity. The LDH@AC composite displayed an enhanced surface area and pore volume than of pristine LDH, suggesting effective integration of LDH onto the AC matrix and enhanced porosity. The BET analysis results indicated that the LDH@AC composite is a promising candidate for catalysis processes, as of its enhanced surface area, porosity, and surface properties.

**Table 1:** Surface area and pore characteristic of different samples.

Samples	BET Surface Area (m^2^/g)	Average pore size (nm)	Pore volume (cm^3^/g)
AC	1097.96	3.444	0.945
NiFe-LDH	103.173	4.553	0.117
NiFe-LDH@AC	543.169	3.383	0.459

### Experimental results of NOR degradation

#### Optimum mass ratio for LDH@AC catalyst

**Fig. 5:**
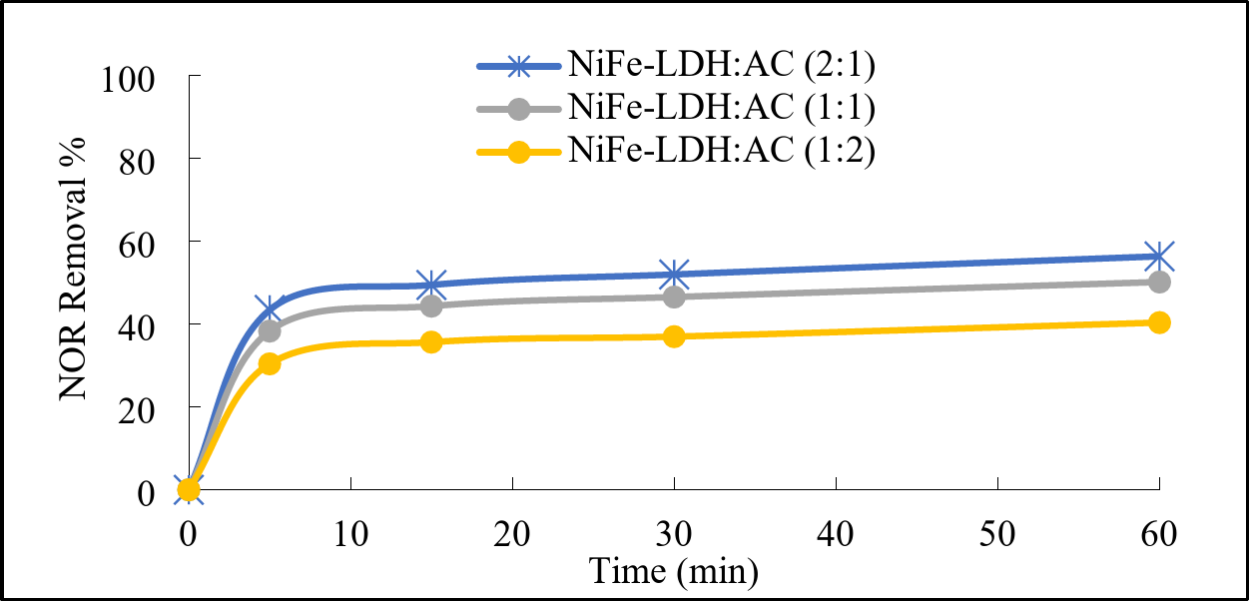
Degradation efficiency of NOR using different mass ratios of LDH@AC composites, Reaction conditions: 100 mL of NOR solution (50 mg/L), 0.1 g/L of sample, 1 g/L of PDS, initial pH 7, and ambient temperature 21°C.

The influence of mass ratios of prepared NiFe-LDH: AC composites were investigated for the activation of PDS and degradation of NOR antibiotic. As shown in Fig. 5, the introduction of both NiFe-LDH@AC samples and PDS simultaneously resulted in moderate degradation efficiencies of NOR. This suggests that the prepared NiFe-LDH@AC catalysts were capable of successfully activating PDS, leading to the generation of oxidative species such as sulfate radicals (SO_4_^•−^) and hydroxyl radicals (^•^OH). The NiFe-LDH@AC catalyst with mass ratio (2:1) showed the highest PDS activation and NOR removal efficiency. This could be interpreted that the ratio of NiFe-LDH to activated carbon affected the surface area and active sites available for the adsorption of NOR and the activation of persulfate^17^. Higher NiFe-LDH content with dispersed on AC sheets might increase the number of active sites for PDS activation, leading to enhanced degradation of NOR^19,32^. As the NiFe-LDH@AC catalyst with mass ratio (2:1) was found to be the optimum composition, so that it was used in the rest of the study and composite characterization.

#### Comparison of adsorption and degradation performances

**Fig. 6:**
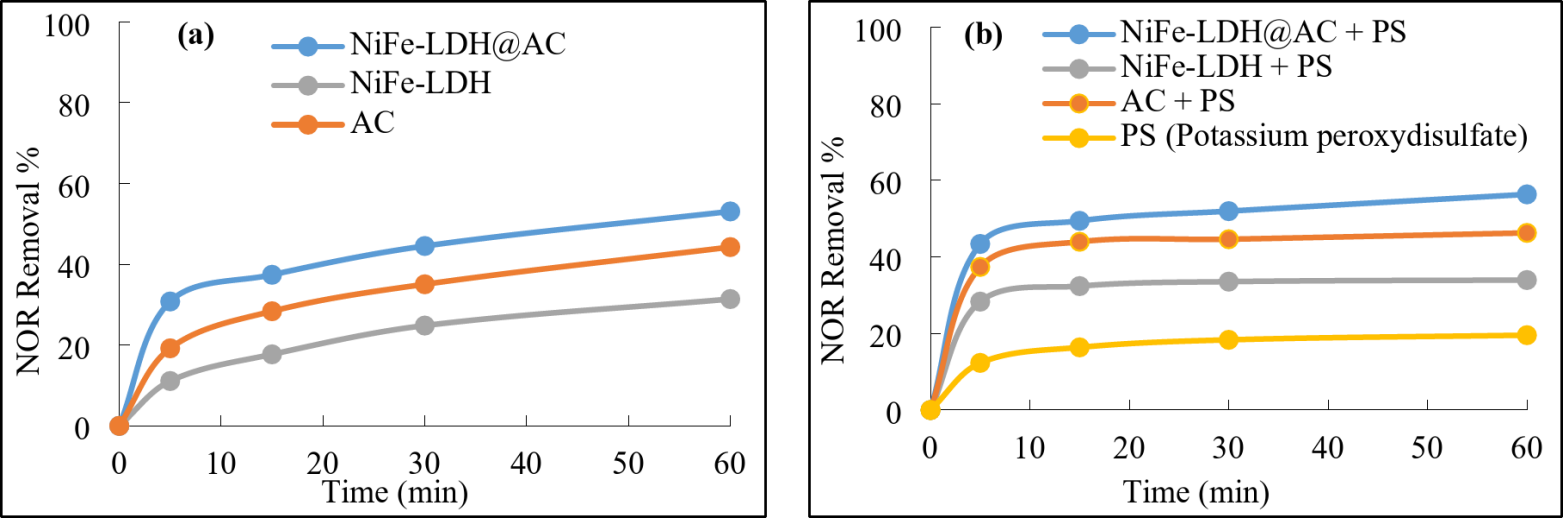
Figure 6 The adsorption efficiency of NOR with different samples (**a**), and the degradation efficiency of NOR with different samples (**b**), Reaction conditions: 100 mL of NOR solution (50 mg/L), 0.1 g/L of sample, 1 g/L of PDS, initial pH 7, and ambient temperature 21°C.

The adsorption and degradation efficiencies were investigated by NOR removal for the different samples with adding the PDS (oxidizing agent) for the latter. As shown in Fig. 6(a), the adsorption performance was highest for the LDH@AC composite with about 53% after 60 min, while bare AC adsorption efficiency was lower with roughly 44%. Therefore, the LDH@AC exhibited better adsorption performance compared to bare AC^20^.

As shown in Fig. 6(b), the degradation performances were in the order of LDH@AC composite > AC > LDH > PDS. The PDS alone showed very low oxidizability (E_0_= 2.1 V), so relying solely on a single chemical oxidant was insufficient for the efficient decomposition of organic pollutants^21^. The AC exhibited good performance for NOR removal with about 46% due to its ability in adsorbing NOR and also activating persulfate to generate radicals^33^. Moreover, the NiFe-LDH composite displayed a lower degradation rate of 34%, which confirmed the composite ability of activating persulfate but not very efficient alone^18^. For the case of LDH@AC composite, the removal rate of NOR reached 56% which was significantly higher compared to the removal rates achieved by each individual component. This suggests the presence of a synergistic catalytic effect between AC and LDH^15^. The porous AC serves as an excellent support matrix, offering a substantial reactive surface area for efficient deposition of LDH while preventing its aggregation^20^. Therefore, the number of active sites available for catalysis was increased and also the specific surface area was enhanced^15^. Moreover, the incorporation of AC has resulted in an increased adsorption capacity of LDH@AC composite for diverse pollutants^17^. Accordingly, this gave more opportunities for the pollutant to come into contact with radical species, leading to a higher degradation efficiency of NOR^19^.

It is crucial to distinguish between adsorption and actual degradation. While adsorption effectively removed NOR from aqueous solutions by transferring it to the adsorbent surface, it did not address the persistence of NOR in the environment, as the pollutant remained chemically intact and could desorb back under certain conditions. This limitation made adsorption unsuitable for long-term environmental remediation^10^. In contrast, the catalytic oxidation process using the NiFe-LDH@AC/PDS system not only removed NOR but also achieved its mineralization into less harmful compounds, such as CO₂, water, and fluoride ions^34^.

#### Effects of reaction parameters on NOR degradation and kinetics

##### Catalyst dose

**Fig. 7:**
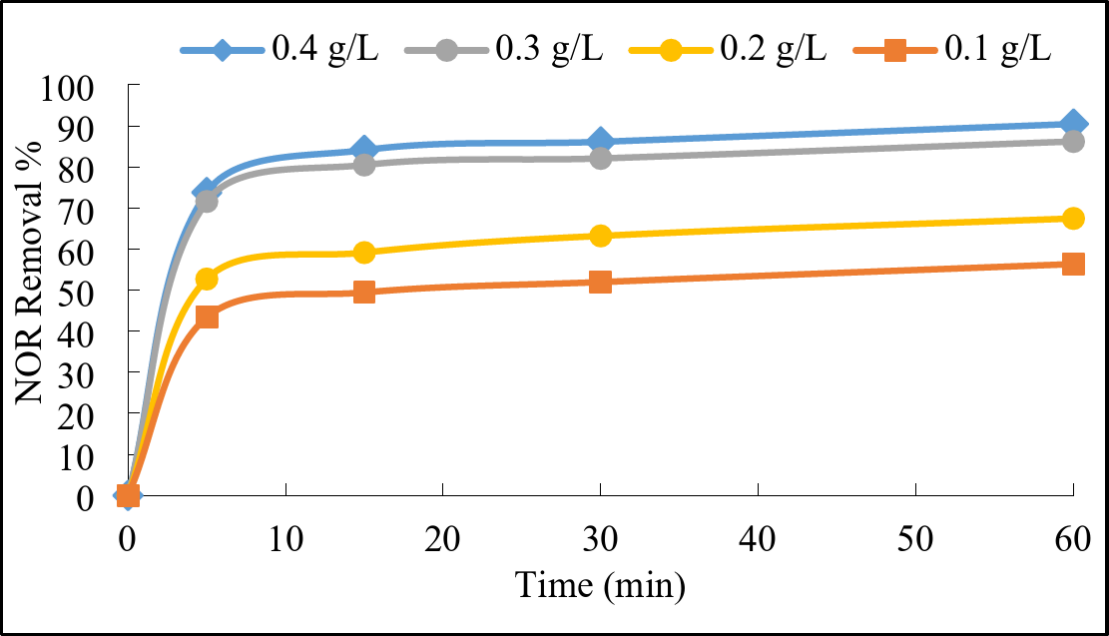
Effect of catalyst dose on the degradation of NOR. Reaction conditions: 100 mL of NOR solution (50 mg/L), 1 g/L of PDS, initial pH 7, and ambient temperature 23°C.

The LDH@AC catalyst dose effect on the removal efficiency of the NOR was studied. As shown in Fig. 7, the increase in the catalyst dosage had a significant effect, as the degradation efficiency was greatly enhanced achieving about 85% removal in the first 15 min. The degradation kinetics (Fig. S1) showed that the reaction rate constant increased from 0.0043 to 0.0163 min.^−1^ with increasing the dose from 0.1 to 0.4 g/L. In fact, the higher catalyst dose typically provided more active sites for PDS activation. Consequently, the activation of PDS is enhanced, resulting in the generation of more sulfate radicals (SO_4_^•−^)^3^. The higher catalyst dose also gave a greater availability of active sites for the catalytic reaction and provided more opportunities for the interaction between NOR molecules and the generated sulfate radicals^19^. Therefore, the degradation efficiency of NOR was improved, as more NOR molecules could be effectively oxidized and decomposed. The 0.4 g/L catalyst dosage achieved about 90% removal efficiency of NOR while the 0.3 g/L attained convergent removal efficiency of about 86%. From economic perspective, the 0.3 g/L dosage was chosen as the operating condition for the subsequent experiments.

##### PDS concentration

**Fig. 8:**
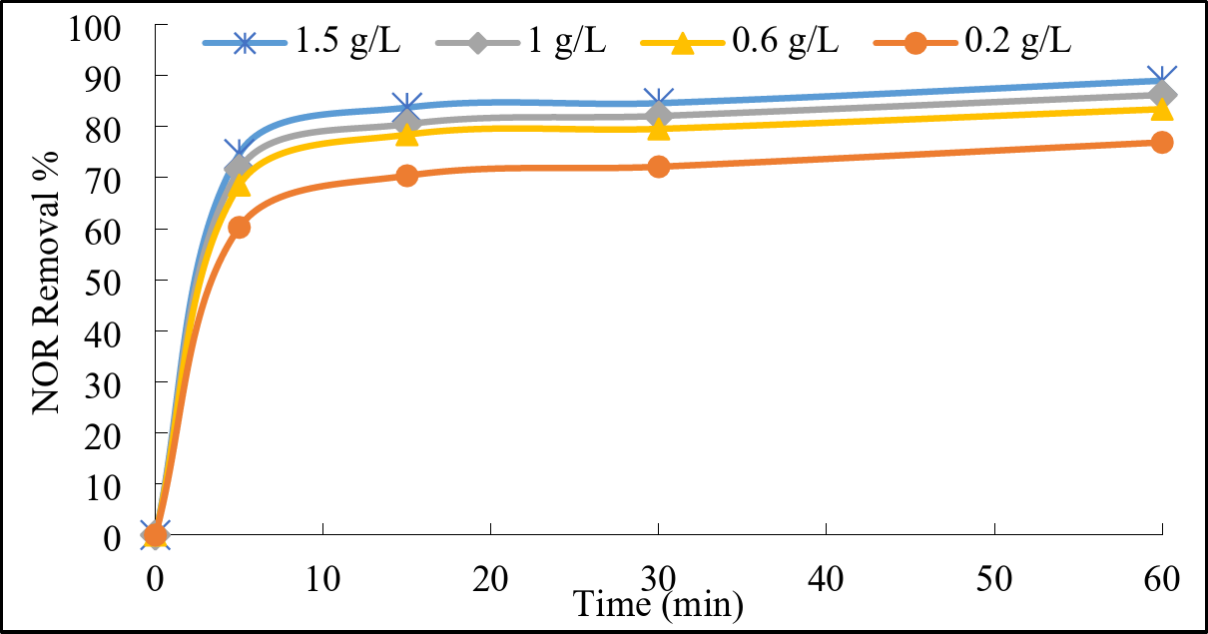
Effect of PDS concentration on the degradation of NOR. Reaction conditions: 100 mL of NOR solution (50 mg/L), 0.3 g/L of catalyst, initial pH 7, and ambient temperature 23°C.

As shown in Fig. 8, the increase in the PDS concentration from 0.2 to 1.5 g/L led to a higher degradation efficiency of NOR and reached 89% at the highest dose. The degradation kinetics analysis (Fig. S1) revealed that the reaction rate constant increased from 0.0086 to 0.0132 min^−1^ with increasing the dose from 0.2 to 1.5 g/L. This could be interpreted that the higher PDS concentrations provided a greater number of PDS molecules in the reaction mixture which accordingly resulted in a higher concentration of sulfate radicals (SO_4_^•−^) generated during PDS activation^35^. The higher oxidation potential of the increased sulfate radicals resulted in more efficient degradation of NOR. Nevertheless, as the PDS concentrations became higher, its effect became weaker on rising the removal efficiency. This could be interpreted that at very high PDS concentrations (1.5 g/L), there might be an excessive generation of radical species, potentially leading to radical scavenging reactions (Eqs. 3 and 4) or the formation of undesired by-products^19,36^. Accordingly, the 1 g/L PDS dose was used as the operating parameter for further experiments.

$$\:{SO}_{4}^{.-}+{SO}_{4}^{.-}\to\:\:{S}_{2}{O}_{8}^{2-}\:\:\:\:\:\:\:\:\:\:\:\:\:\:\:\:\:\:\:\:\:\left(3\right)$$

$$\:{SO}_{4}^{.-}+{S}_{2}{O}_{8}^{2-}\to\:\:S{O}_{4}^{2-}+\:{S}_{2}{O}_{8}^{.-}\:\:\:\:\left(4\right)$$

##### Initial pH value

**Fig. 9:**
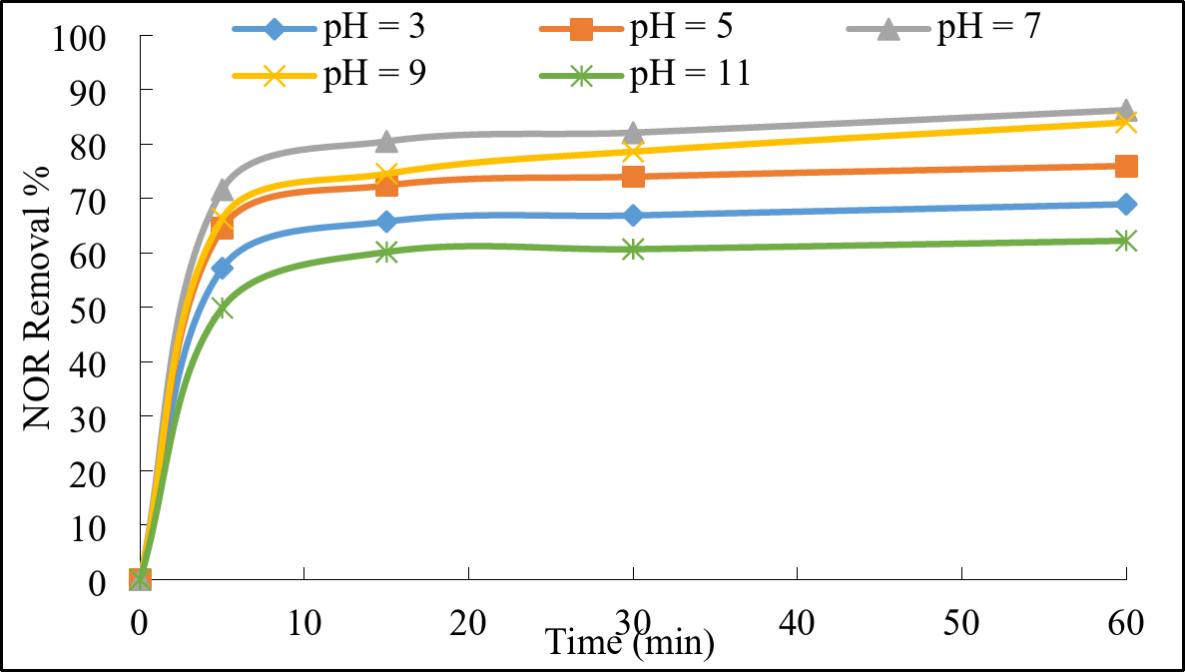
Effect of initial pH value on the degradation of NOR. Reaction conditions: 100 mL of NOR solution (50 mg/L), 0.3 g/L of catalyst, 1 g/L of PDS, and ambient temperature 23°C.

The pH value effect on the removal efficiency of the NOR was studied as shown in Fig. 9. The pH value had a significant effect on the speciation of both the persulfate and NOR, as well as the surface charge and reactivity of the catalyst^19^. The neutral pH value showed the highest degradation efficiency with 86.2%, then the pH value of 9 showed a slightly lower removal efficiency with 84%. The removal efficiency decreased with high acidic and alkaline conditions to about 69% and 62%, respectively. This could be interpreted that at neutral pH, the persulfate activation was more efficient compared to extremely acidic or basic conditions. As, the equilibrium between different persulfate species (such as S_2_O_8_²⁻, HSO_5_⁻, SO_5_²⁻) favored the generation of sulfate radicals, enhancing the degradation process^3,37^. Moreover, NOR is a weak basic compound with 2 pK_a_ values of pK_a1_ = 6.3 and pK_a2_= 8.5, so at neutral pH, it was predominantly in its zwitterionic species with zero net charge, which typically exhibited higher reactivity towards oxidative degradation processes^38,39^. Furthermore, the NiFe-LDH@AC catalyst had the point of zero charge at 6.8 which exhibited optimal stability and activity under neutral pH conditions^40^. Extreme acidic pH values could lead to the leaching of active components or structural degradation of the catalyst, reducing its effectiveness^21^. Neutral pH provided a balanced environment that maintains catalyst integrity while promoting efficient norfloxacin degradation which is compatible with previous literature^3^.

##### NOR initial concentration

**Fig. 10:**
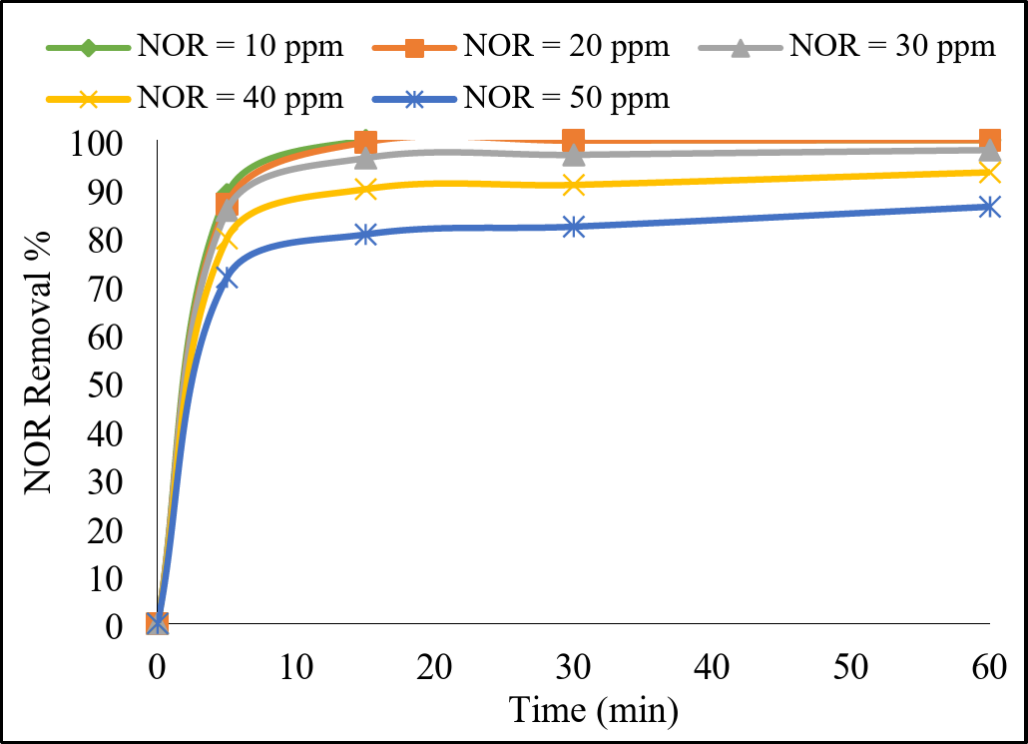
Effect of NOR initial concentration on its degradation rate. Reaction conditions: 0.3 g/L of catalyst, 1 g/L of PDS, initial pH 7, and ambient temperature 23°C.

The NOR initial concentration had an obvious effect on its degradation efficiency as shown in Fig. 10. The NOR degradation efficiency decreased from 100 to 86% when increasing NOR initial concentration from 10 to 50 mg/L, respectively. This could be interpreted that as the concentration of NOR increases, the available active sites on the catalyst surface might become more saturated quickly, limiting further degradation. It might also result in increased competition for the available reactive species, such as sulfate radicals, leading to a decrease in the overall degradation efficiency^3^. On the contrary, at lower initial concentrations there might be abundance of active sites on the catalyst surface for adsorption and subsequent degradation of NOR molecules^2^. Furthermore, the degradation kinetics analysis (Fig. S1) revealed that the reaction rate constant highly increased from 0.0116 to 0.502 min^−1^with decreasing the initial concentration from 50 to 10 mg/L. These results confirmed that kinetics of NOR degradation could be influenced by factors such as mass transfer limitations and the availability of reactive species^19^. These results were consistent to the previous studies^41,42^.

##### Reaction temperature

**Fig. 11:**
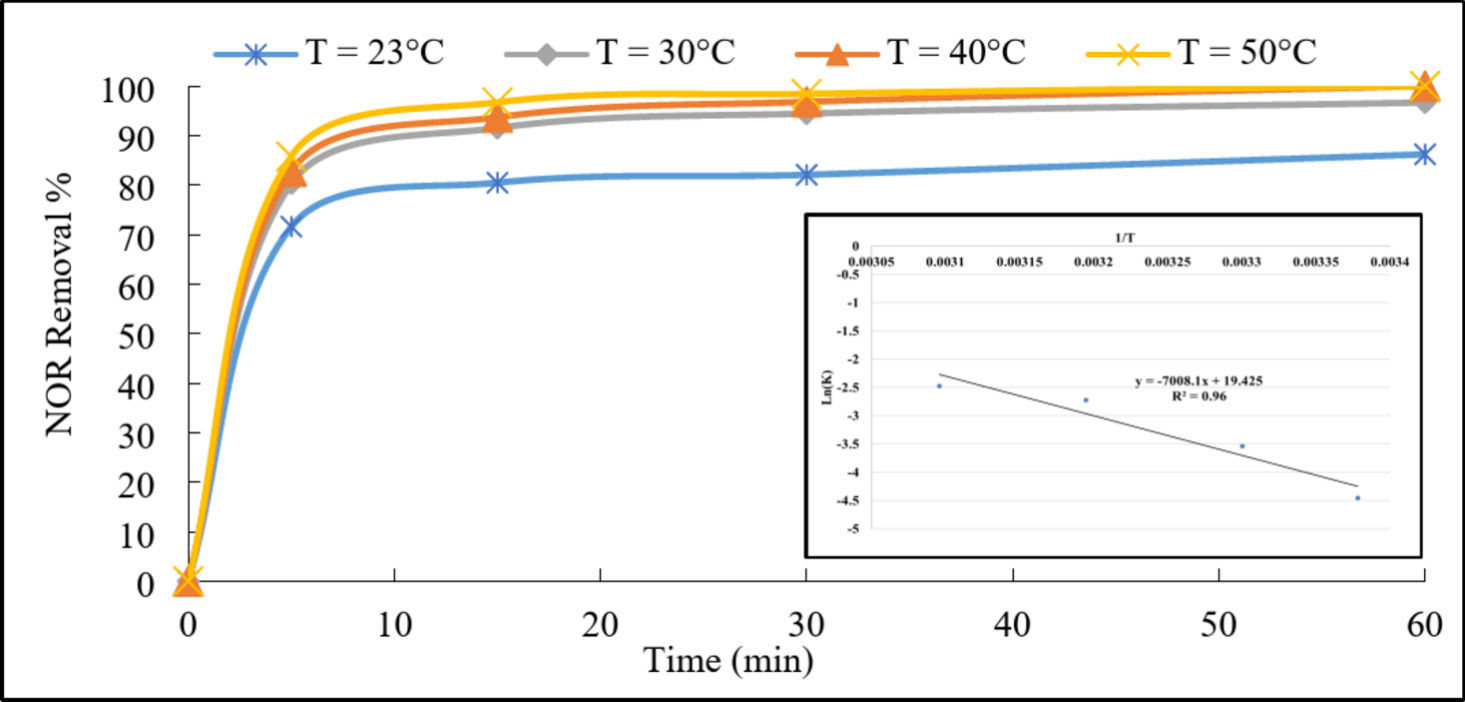
Effect of reaction temperature on the degradation of NOR. Reaction conditions: 100 mL of NOR solution (50 mg/L), 0.3 g/L of catalyst, 1 g/L of PDS, and initial pH 7, (Arrhenius equation fitting in the inset figure).

The ambient temperature effect was studied on the removal efficiency of the NOR as shown in Fig. 11. The degradation efficiency increased from 86 to 100%, when rising the temperature from 23 to 50 ^o^C, respectively. Besides, the degradation kinetics analysis (Fig. S1) revealed that the reaction rate constant increased from 0.0116 to 0.0842 min^−1^ with increasing the temperature from 23 to 50 ^o^C, respectively. This could be interpreted that higher temperature provided more thermal energy to the system, leading to increased molecular motion and collision frequency. This promoted the activation of PDS using LDH catalyst and enhanced the generation of reactive sulfate radicals. Consequently, the degradation of NOR became faster at higher temperatures and these results were consistent with previous literature for NOR degradation^27,43^. Furthermore, the activation energy was evaluated from Arrhenius equation (Eq. 2). The relation between Ln (K) and 1/T was plotted (the inset of Fig. 11) and the E_a_in this system was estimated as 58.27 kJ/mol, consistent with the previously reported PDS and LDH based system for degradation of organics^44^. This activation energy for the degradation process was higher than the typical activation energy range for diffusion-controlled reactions (10 ~ 13 kJ/mol)^19^. This suggests that the degradation was primarily driven by the inherent chemical reactions occurring on the LDH@AC catalyst surface, rather than being limited by mass transfer or diffusion processes^43^.

### ROS determination and activation mechanism analysis

To investigate the role of reactive oxygen species (ROS) in the degradation of NOR, multiple radical scavengers were added to the Ni-Fe LDH@AC catalyst system activated by PDS. According to previous studies, SO₄˙⁻ and HO˙ were the primary radicals in the PDS activation system^19,21^. MeOH reacts readily with both SO_4_˙⁻ and HO˙ radicals^31^. This makes it a broad-spectrum scavenger. TBA has a high reaction rate constant with HO˙ radicals but a much lower reaction rate constant with SO_4_˙⁻ radicals^19^. This selective reactivity makes TBA a useful scavenger to specifically target HO˙.

**Fig. 12:**
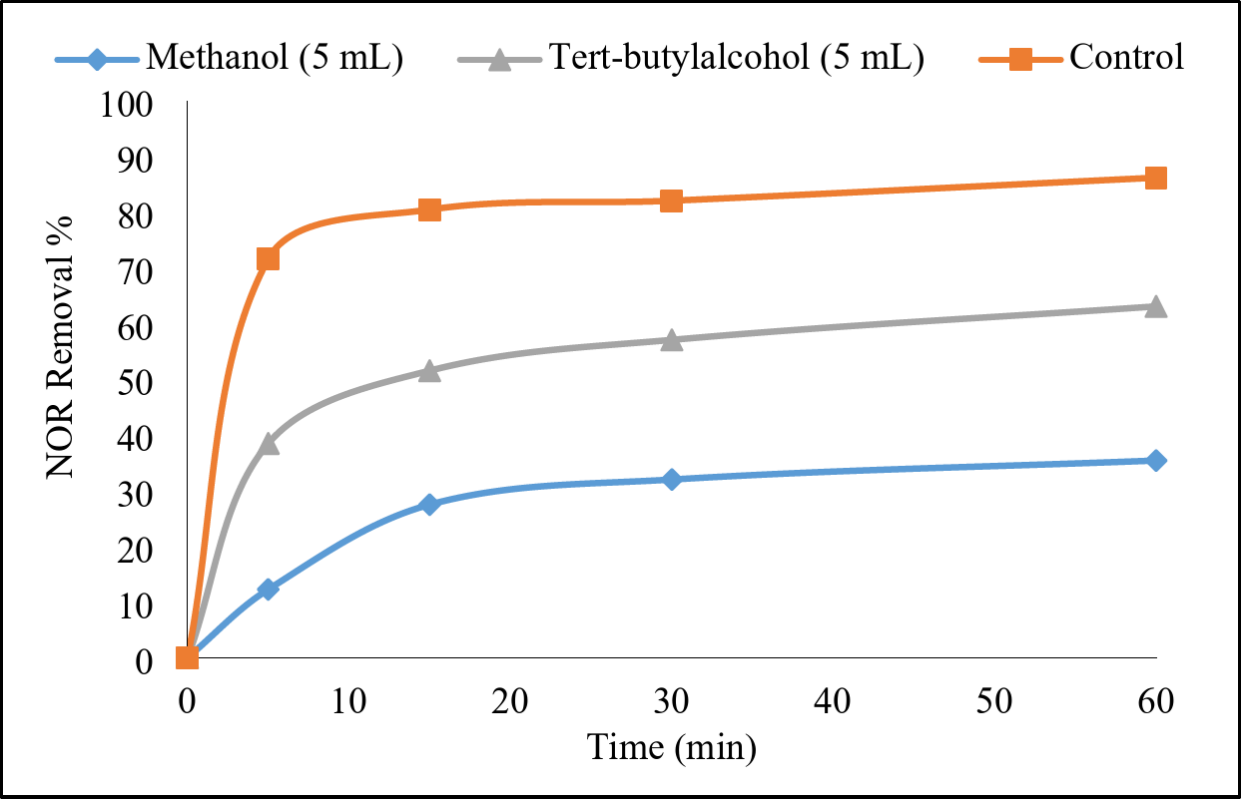
The radicals scavenging experiments. Reaction conditions: 100 mL of NOR solution (50 mg/L), 0.3 g/L of catalyst, 1 g/L of PDS, initial pH 7, and ambient temperature 23°C.

The removal efficiency of NOR, as shown in Fig. 12, decreased within 60 min from 86 to 63% when TBA was added, and to 35% when MeOH was added. This confirmed that the oxidation process involved radical species. Moreover, the addition of MeOH significantly suppressed the degradation of NOR, while adding TBA only caused a slight decrease. Based on these results, it could be rationally inferred that both sulfate and hydroxyl radicals contributed to NOR degradation, but the sulfate radicals played a larger role. This was further supported by literature that has also stated sulfate radicals as the predominant radical species in metal-based persulfate activation systems^3,21^, corroborating the findings of the present study.

**Fig. 13:**
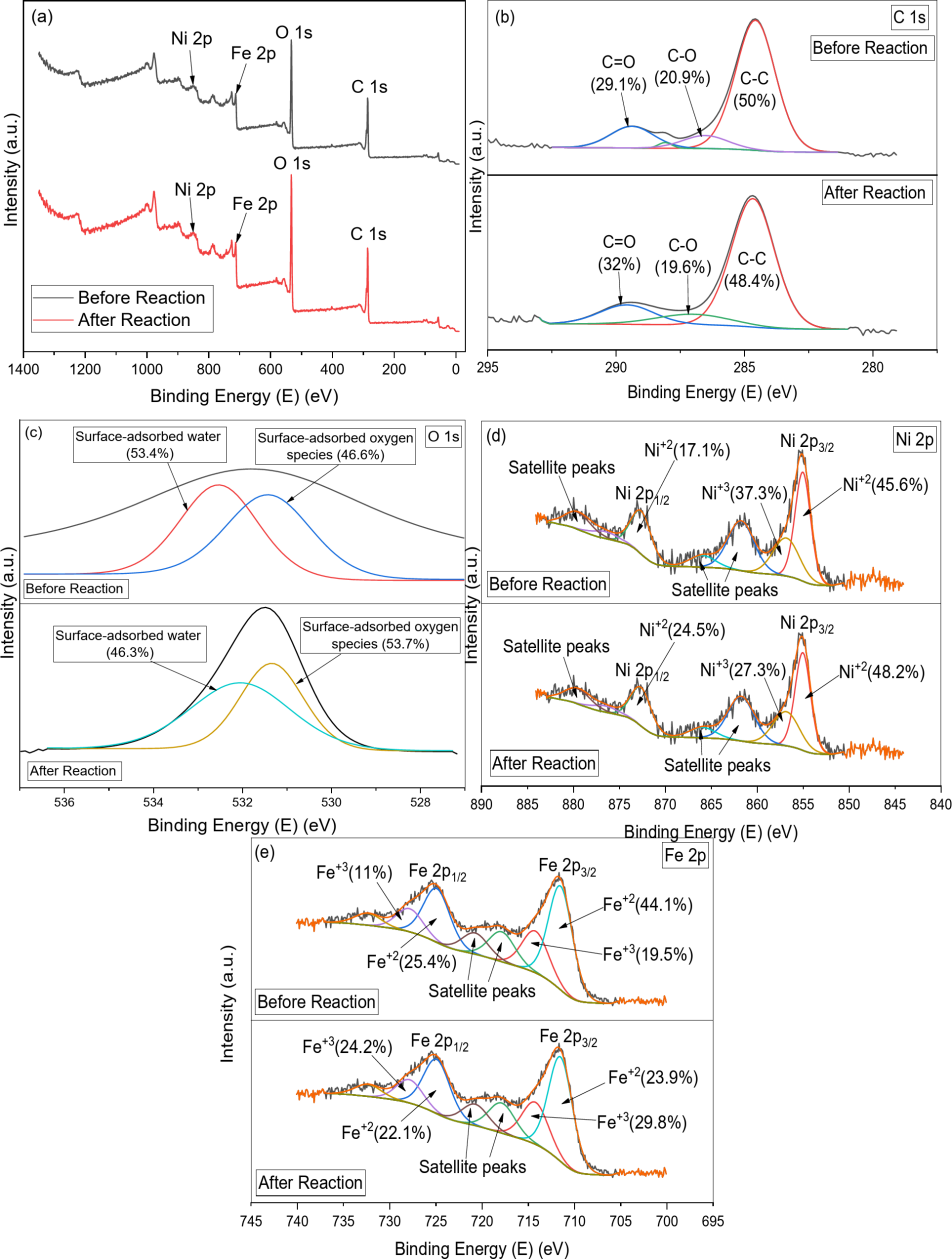
The XPS spectra of NiFe-LDH@AC before and after the reaction for (**a**) XPS wide survey, (**b**) C1s, (**c**) O1s (**d**) Ni2p, and (**e**) Fe2p.

For the study of the mechanism of persulfate activation by NiFe-LDH@AC, the comparative XPS spectra of both fresh and used catalysts were performed and presented in Fig. 13. The XPS wide survey spectra (Fig. 13a) indicated the existence of Ni, Fe, O, and C elements on the surface of NiFe-LDH@AC. In the XPS spectra of C 1s in the NiFe-LDH@AC composite (Fig. 13b), three fitted peaks were detected at 284.6 eV, 286.6 eV, and 289.4 eV. These peaks corresponded to the presence of C–C, C–O, and C = O or O–C = O, respectively^28^. The carbon structure remained mostly stable during the reaction, with slight oxidation indicated by an increase in C = O bonds, likely due to interactions with oxygen-containing species (SO_4_˙⁻ radicals) and NOR intermediates^31^. The XPS spectra of O 1s peak was deconvoluted into two distinct peaks (Fig. 13c); surface-adsorbed oxygen species at 531.4 eV and surface-adsorbed water at 532.5 eV. After the degradation reaction, the surface-adsorbed oxygen species increased from 46.6 to 53.7% due to the presence of adsorbed persulfate (PDS) or NOR intermediates on the surface of the NiFe-LDH@AC composite^31^.

The Ni 2p spectra showed in, Fig. 13d, two primary peaks corresponding to the Ni 2p₃/₂ and Ni 2p₁/₂ levels, along with satellite peaks, indicating the oxidation states of Ni. The peaks at binding energies of 855 eV and 872.8 eV were associated with Ni^+2^, while the peak at 856.9 eV corresponded to Ni^+3^^31^. After the reaction, the Ni^+2^ increased from 62.7 to 72.7%, while Ni^+3^ decreased from 37.3 to 27.3%, respectively. This suggested that Ni^2^⁺ oxidation to Ni^+3^played a significant role in the persulfate activation mechanism, and its reduction to Ni²⁺ was part of the catalytic cycle for continuous generation of ROS from PDS^18^. The Fe 2p spectra in Fig. 13e showed both Fe²⁺ and Fe³⁺ oxidation states, with peaks for Fe 2p₃/₂ and Fe 2p₁/₂ visible, along with satellite features typical of transition metal oxides. Four fitted peaks were detected at which the peaks 711.6 eV and 725 eV were associated with Fe²⁺, while the peaks 714.4 eV, and 728 eV corresponded to Fe^+3^^18^. After the reaction, the Fe²⁺ decreased from 69.5 to 46%, while Fe³⁺ increased from 30.5 to 54%, respectively. The increase in Fe³⁺ after the reaction indicated that Fe²⁺ was oxidized during persulfate activation, confirming the involvement of iron in the redox mechanism of PDS activation^18,31^.

Overall, the XPS analysis revealed that the NiFe-LDH@AC composite experienced significant redox changes during persulfate activation, involving the oxidation states of Ni²⁺/Ni³⁺ and Fe²⁺/Fe³⁺ in the catalytic process. Additionally, slight alterations in the oxygen and carbon environments were observed, likely due to interactions with persulfate radicals.

### Stability and reusability of catalyst

#### Metals dissolution under different pH conditions

**Fig. 14:**
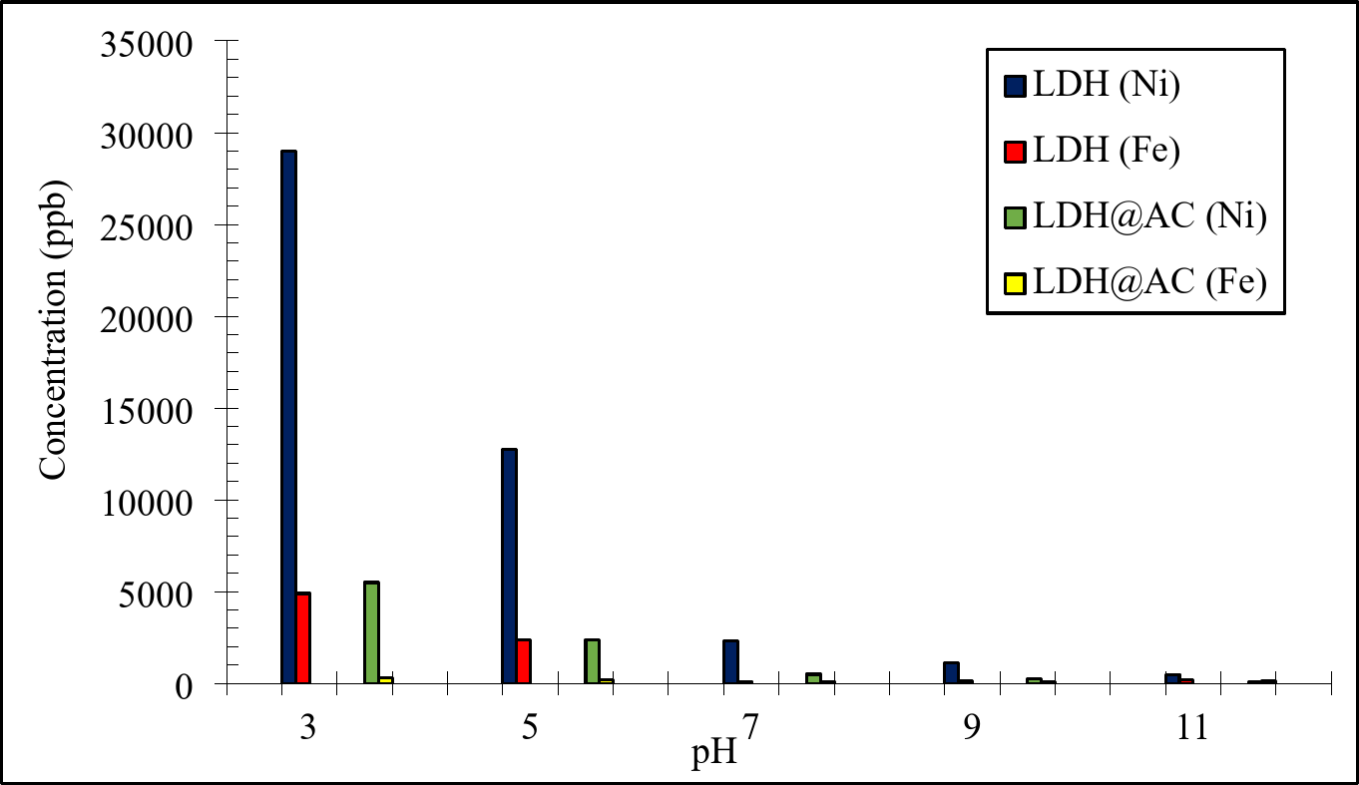
The heavy metals concentration of LDH and LDH@AC in different pH solution after reaction. Reaction conditions: 100 mL of NOR solution (50 mg/L), 0.3 g/L of catalyst, 1 g/L of PDS, initial pH 7, and ambient temperature 23°C.

The concentration of dissolved metals was analyzed using ICP-OES in solutions with different pH values. As shown in Fig. 14, the concentration of dissolved metals was lower in alkaline solutions compared to acidic solutions. This could be interpreted that at acidic conditions, H^+^ ions broke down the hydroxide layer of LDH composite, leading to increased leaching of metals. In contrast, under alkaline conditions, the prevalent OH^−^ions helped to protect the hydroxide layer and stabilize the structure of LDH^21^. Moreover, the Ni ions seemed to have higher solubility than the iron ions specifically in acidic conditions. This could be explained that the solubility of Ni compounds, particularly NiO, in acidic conditions is relatively high, leading to a significant concentration of Ni²⁺ ions in solution^45^. However, iron oxides do dissolve in acids, but they tend to do so less readily than Ni oxide. The formation of various iron complexes in solution can also influence their overall solubility^46^.

Notably, when comparing the LDH system to the LDH@AC system, the concentration of dissolved metals decreased significantly in the LDH@AC system. Specifically, the decreases were 83% at pH 3.0, pH 5.0, 76% at pH 7.0, 71% at pH 9.0, and 70% at pH 11.0. This indicated that the activated carbon support enhanced the structural stability of the LDH, reducing metal leaching^21^. The results demonstrated that LDH@AC catalysts were more stable across a wider range of pH conditions, which is advantageous for practical applications in wastewater treatment where pH can vary.

#### Reusability of LDH@AC composite

**Fig. 15:**
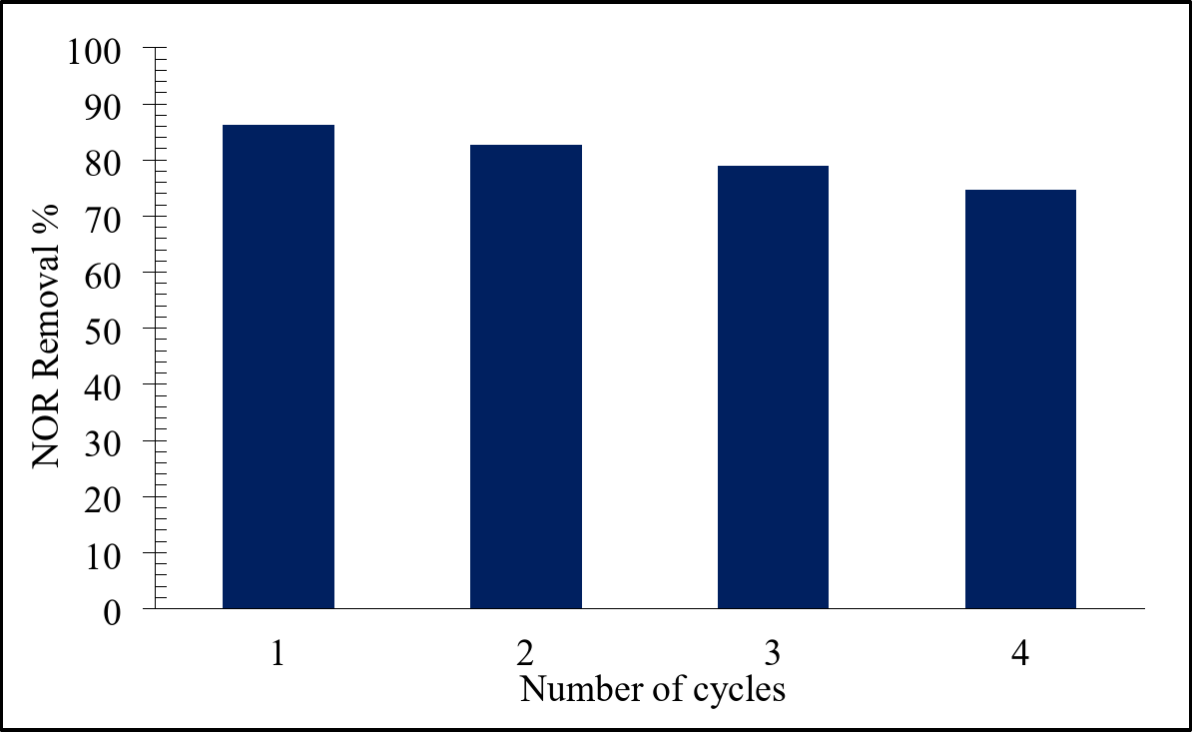
The changes of degradation efficiency within four consecutives degradation runs. Reaction conditions: 100 mL of NOR solution (50 mg/L), 0.3 g/L of catalyst, 1 g/L of PDS, initial pH 7, and ambient temperature 23°C.

It was essential to take into account the reusability and stability of the catalyst, as for its probable use in wastewater treatment. Therefore, reusability experiments were conducted over four consecutive runs under identical conditions. As shown in Fig. 15, the catalytic performance of NiFe-LDH@AC slightly declined with each successive run, likely due to the inability to fully recover and reuse all of the NiFe-LDH@AC catalyst material^10^and also the loss of Ni and Fe ions along the successive runs might be a reason^18^. Despite this, about 75% NOR degradation efficiency was achieved in the fourth run, demonstrating that LDH@AC maintained good reusability overall.

This suggested that NiFe-LDH@AC is a promising material for successive usage in activating persulfate for the degradation of contaminants like NOR in wastewater treatment applications. Further studies focusing on regenerating the catalyst or optimizing its composition could help mitigate the decline in activity observed over multiple cycles.

### Degradation pathways of NOR

**Fig. 16:**
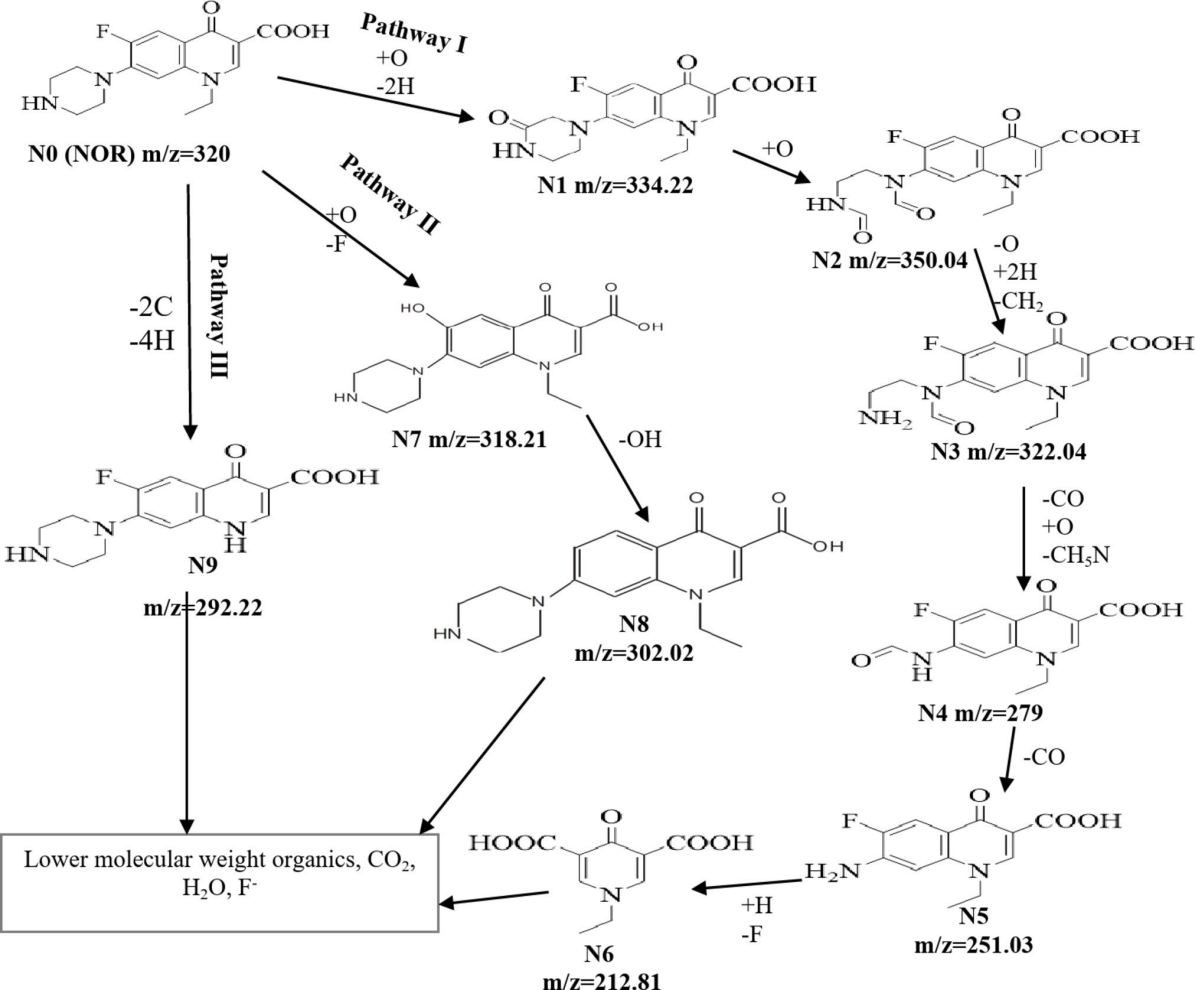
The proposed degradation pathways of NOR in NiFe-LDH@AC/PDS system.

The proposed degradation pathways of NOR in the PDS activation by NiFe-LDH@AC system (Fig. 16) were identified using LC-ESI-MS/MS analysis and previous related studies^3,6,27,41,47^. The analysis involved the identification of nine intermediate products, labeled as N1 to N9, during the oxidation of NOR (Fig. S3). These intermediates were summarized showing their respective mass-to-charge ratios (m/z), retention times, and possible molecular structures, as detailed in Table S1. The NOR molecule had a structure composed of a benzene ring and a piperazine ring, which were the regions with the highest electron density and the most vulnerable to be attacked by free radicals, such as sulfate radicals^6^. Three degradation pathways - piperazine ring cleavage, defluorination, and dealkylation were identified as the primary mechanisms by which sulfate radicals facilitate the transformation and mineralization of the NOR compound^27^.

In Pathway I, the Piperazine ring cleavage achieved through a sequence of reactions starting with oxidation and dehydrogenation to form N1 and N2. A series of modifications occurred including demethylation and decarbonylation, forming intermediates N3 to N5. The pathway concluded with hydrodefluorination reaction, resulting in N6^27,41^.

In Pathway II, the NOR encountered defluorination and oxidation reactions to form N7. N7 then underwent further transformation by dehydroxylation reaction, producing N8^6,47^. This pathway focused on the removal of fluorine and subsequent structural changes.

In Pathway III, NOR underwent deethylation, removing an ethyl group from the quinolone ring, which involved losing two carbon atoms and four hydrogen atoms, resulting in the formation of N9^27^. This pathway highlighted significant structural changes due to the removal of larger alkyl groups.

The degradation of NOR through these pathways ultimately resulted in the formation of lower molecular weight organic compounds, including carbon dioxide (CO_2_), water (H_2_O), and fluoride ions (F^−^), signifying the complete breakdown of the original molecule into simpler components under continuous attack by SO_4_^•−^and •OH radicals^48^.

In previous studies, Liu et al.^49^ used a sewage sludge-derived char-based catalyst with H₂O₂ generated •OH for NOR degradation, identifying eight oxidative intermediates through hydroxylation and decarboxylation including intermediates like quinolone derivatives and open-ring structures​. Similarly, Diao et al.^50^ reported that a nanoscale zero-valent iron (nZVI) catalyst with ultrasound enhanced NOR breakdown into simpler organic acids and alcohols via •OH radicals​. Compared to these methods, the NiFe-LDH@AC catalyst exhibited superior stability and reusability while achieving efficient NOR degradation. Unlike the other systems, the integration of layered double hydroxides (LDHs) with activated carbon provided a synergistic platform, enhancing both the generation of SO_4_^•−^ and catalyst stability.

### Reaction mechanism for NOR degradation

In NiFe-LDH@AC/PDS system, the activation of PDS by the NiFe-LDH@AC composite involved a series of redox reactions and radical generation steps. Initially, the transition metals (Ni²⁺ and Fe²⁺) on the catalyst surface donated electrons to activate PDS by breaking its peroxide bond (O-O) bond, producing highly reactive sulfate radicals (SO_4_^•−^) and hydroxyl ions (OH⁻) (Eqs. 5 and 6)^18,51^. The generated SO_4_^•−^radicals underwent further reactions to produce additional reactive oxygen species (ROS), such as hydroxyl radicals (•OH), which contributed to the oxidative breakdown of NOR (Eq. 7)^19,51^. The ROS generated in the system attacked NOR molecules, leading to the cleavage of the piperazine ring and defluorination of the aromatic structure, forming intermediate products such as carboxylic acids, which eventually mineralized into CO₂ and H₂O (Eq. 8)^19^.

A critical feature of this catalytic system is the Ni²⁺/Ni³⁺ and Fe²⁺/Fe³⁺ redox cycles, which regenerated the active metal sites to sustain continuous PDS activation (Eq. 9)^51^. Fe³⁺ and Ni³⁺ were further reduced by interactions with PDS, closing the catalytic loop (Eqs. 10 and 11)^18,51^. The activated carbon (AC) in the composite played a crucial role by providing a high surface area for PDS adsorption and enhancing electron transfer. This synergistic effect not only boosted ROS generation but also reduced the leaching of metal ions, ensuring catalyst stability^18^. This comprehensive mechanism highlighted the effectiveness of NiFe-LDH@AC in activating PDS, generating ROS, and achieving efficient NOR degradation.

$$\:{Ni}^{2+}+{HS{O}_{5}}^{-}\to\:\:{Ni}^{3+}+{S{O}_{4}}^{{\bullet\:}-}+{OH}^{-}\:\:\:\:\:\:\:\:\:\:\:\:\:\:\:\left(5\right)$$

$$\:{Fe}^{2+}+{HS{O}_{5}}^{-}\to\:\:{Fe}^{3+}+{S{O}_{4}}^{{\bullet\:}-}+{OH}^{-}\:\:\:\:\:\:\:\:\:\:\:\:\:\:\:\left(6\right)$$

$$\:{S{O}_{4}}^{{\bullet\:}-}+{H}_{2}O\to\:\:{H}^{+}+{S{O}_{4}}^{2-}+{\bullet\:}\text{O}\text{H}\:\:\:\:\:\:\:\:\:\:\:\:\:\:\:\:\:\:\:\:\:\left(7\right)$$

$$\:{S{O}_{4}}^{{\bullet\:}-}+{\bullet\:}\text{O}\text{H}+\text{N}\text{O}\text{R}\to\:\:C{O}_{2}+{H}_{2}O\:\:\:\:\:\:\:\:\:\:\:\:\:\:\:\:\:\:\:\:\:\:\left(8\right)$$

$$\:{Ni}^{3+}+{Fe}^{2+}\to\:\:{Ni}^{2+}+{Fe}^{3+}\:\:\:\:\:\:\:\:\:\:\:\:\:\:\:\:\:\:\:\:\:\:\:\:\:\:\:\:\:\:\:\:\:\:\left(9\right)$$

$$\:{Ni}^{3+}+{HS{O}_{5}}^{-}\to\:\:{Ni}^{2+}+{S{O}_{5}}^{{\bullet\:}-}+{H}^{+}\:\:\:\:\:\:\:\:\:\:\:\:\:\:\:\:\left(10\right)$$

$$\:{Fe}^{3+}+{HS{O}_{5}}^{-}\to\:\:{Fe}^{2+}+{S{O}_{5}}^{{\bullet\:}-}+{H}^{+}\:\:\:\:\:\:\:\:\:\:\:\:\:\:\:\:\left(11\right)$$

### Real application study

As a real application, a treated wastewater sample mixed with NOR solution was experimented in the NiFe-LDH@AC/PDS system. The results showed that the NOR degradation efficiency reached 84%, which was very close to 86% achieved in DI water. Moreover, the measured COD decreased from 76 mg/L to 34 mg/L achieving a removal efficiency of 55% indicating satisfactory progress in the mineralization of organic contaminants, including NOR as a target pollutant^3^. These findings demonstrated that the catalytic degradation is successful in achieving good results in real applications.

## Conclusion

The NiFe-LDH@AC composite demonstrated high efficiency in activating persulfate for the catalytic degradation of Norfloxacin (NOR) in aqueous systems. Characterization studies confirmed the successful synthesis and structural stability of the composite, with a significant improvement in surface area and active site availability. The catalyst exhibited excellent performance across various reaction parameters, including pH, PDS dosage, and temperature, achieving optimal degradation efficiency at neutral pH and high temperatures. XPS analysis revealed significant redox changes in Ni²⁺/Ni³⁺ and Fe²⁺/Fe³⁺ during persulfate activation, supporting the proposed redox mechanism. The composite also showed good reusability over multiple cycles, with minimal metal leaching. The LC-ESI-MS/MS analysis identified multiple degradation pathways for NOR, confirming the complete breakdown of the antibiotic into smaller, less harmful products. The real application study confirmed the practical effectiveness of the NiFe-LDH@AC/PDS system for environmental remediation. Overall, NiFe-LDH@AC is a highly effective and stable catalyst for PDS activation and NOR degradation. Its high catalytic activity, stability, and minimal environmental impact make it a promising candidate for the treatment of pharmaceutical contaminants in wastewater. Further studies could explore optimizing the catalyst composition and extending its application to other recalcitrant pollutants.

## Electronic supplementary material

Below is the link to the electronic supplementary material.

Supplementary Material 1

## Data Availability

Data is provided within the manuscript and supplementary information file.
