# Supplementary material for: Catalytic degradation of norfloxacin using persulfate activation by Ni-Fe layered double hydroxide catalyst supported on activated carbon
Source: Sci Rep. 2025 Feb 11;15:5132. doi: 10.1038/s41598-025-89106-w (PMC11814233; doi:10.1038/s41598-025-89106-w)
Supplement: Supplementary file 1 — Supplementary Material 1 [file 41598_2025_89106_MOESM1_ESM.docx]

Catalytic Degradation of Norfloxacin using Persulfate Activation by Ni-Fe Layered Double Hydroxide Catalyst Supported on Activated Carbon

Adel Adly^1,*^, Mona M Galal^2^, Minerva E Matta^2^

^1^Dar Engineering Consultancy, Smart Village, Giza 12577, Egypt (Corresponding author)

^2^Sanitary and Environmental Engineering Division, Faculty of Engineering, Cairo University, 12613 Giza, Egypt.

^*^Corresponding Author. (Email: [eng.adeladly91@gmail.com](mailto:eng.adeladly91@gmail.com))

**Kinetics (pseudo-first-order model)**

Figure S1 The estimated values of reaction rate constants for various reaction parameters of NOR degradation using persulfate activation by Ni-Fe LDH@AC catalyst

|  |  |
| --- | --- |
|   Figure S2 N_2_ adsorption-desorption isotherms of (a) AC, (b) NiFe-LDH, (c) NiFe-LDH@AC,  and the corresponding pore size distributions (inset). | |

Figure S3 The mass spectra of degradation intermediate products of NOR in NiFe-LDH@AC/PS system detected by LC-ESI-MS

N0

N1

N2

N3

N4

N5

N6

N7

N8

N9

Table S1 The detailed information of the NOR degradation intermediates detected by LC-ESI-MS/MS analysis

| **Compound No.** | **Retention time (min)** | **Formula** | **m/z (Da)** | **Exact mass (Da)** | **Proposed structure** |
| --- | --- | --- | --- | --- | --- |
| N0 (NOR) | 8.31 | C_16_H_18_FN_3_O_3_ | 320.01 | 319.01 | 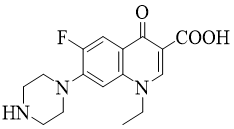 |
| N1 | 13.28 | C_16_H_16_FN_3_O_4_ | 334.22 | 333.22 | 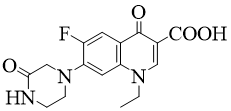 |
| N2 | 7.34 | C_16_H_16_FN_3_O_5_ | 350.04 | 349.04 | 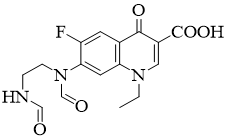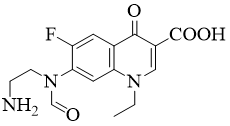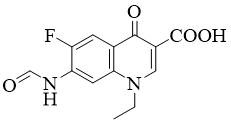 |
| N3 | 4.19 | C_15_H_16_FN_3_O_4_ | 322.04 | 321.04 |  |
| N4 | 7.76 | C_13_H_11_FN_2_O_4_ | 279 | 278 |  |
| N5 | 9.15 | C_12_H_11_FN_2_O_3_ | 251.03 | 250.03 | 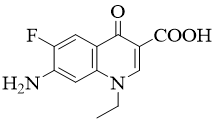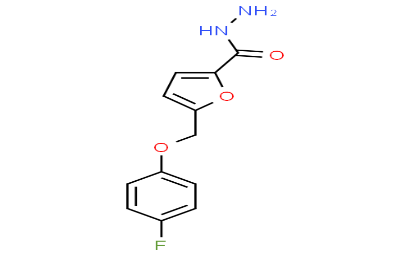 |
| N6 | 1.05 | C_12_H_12_N_2_O_3_ | 212.81 | 211.81 | 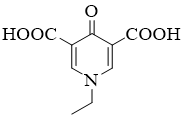 |
| N7 | 13.41 | C_16_H_19_N_3_O_4_ | 318.21 | 317.21 | 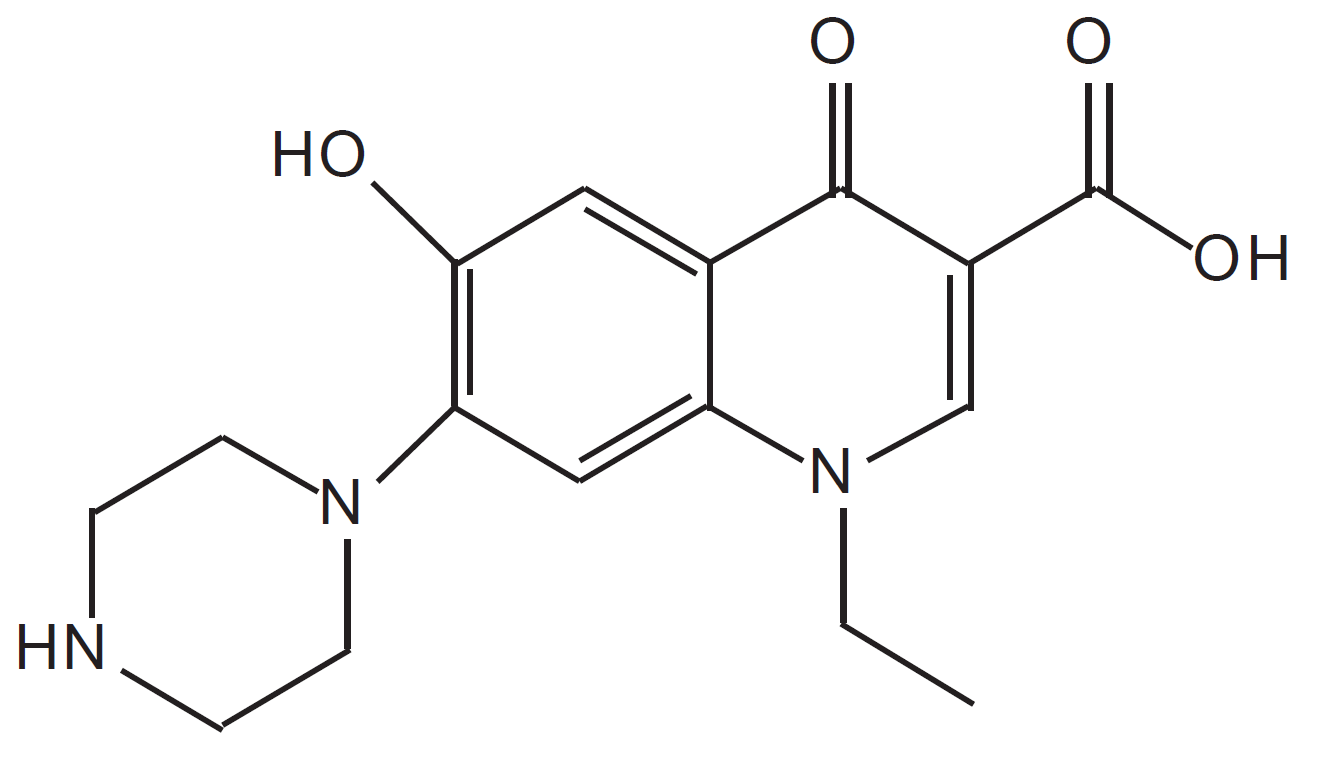 |
| N8 | 6.87 | C_16_H_19_N_3_O_3_ | 302.02 | 301.02 | 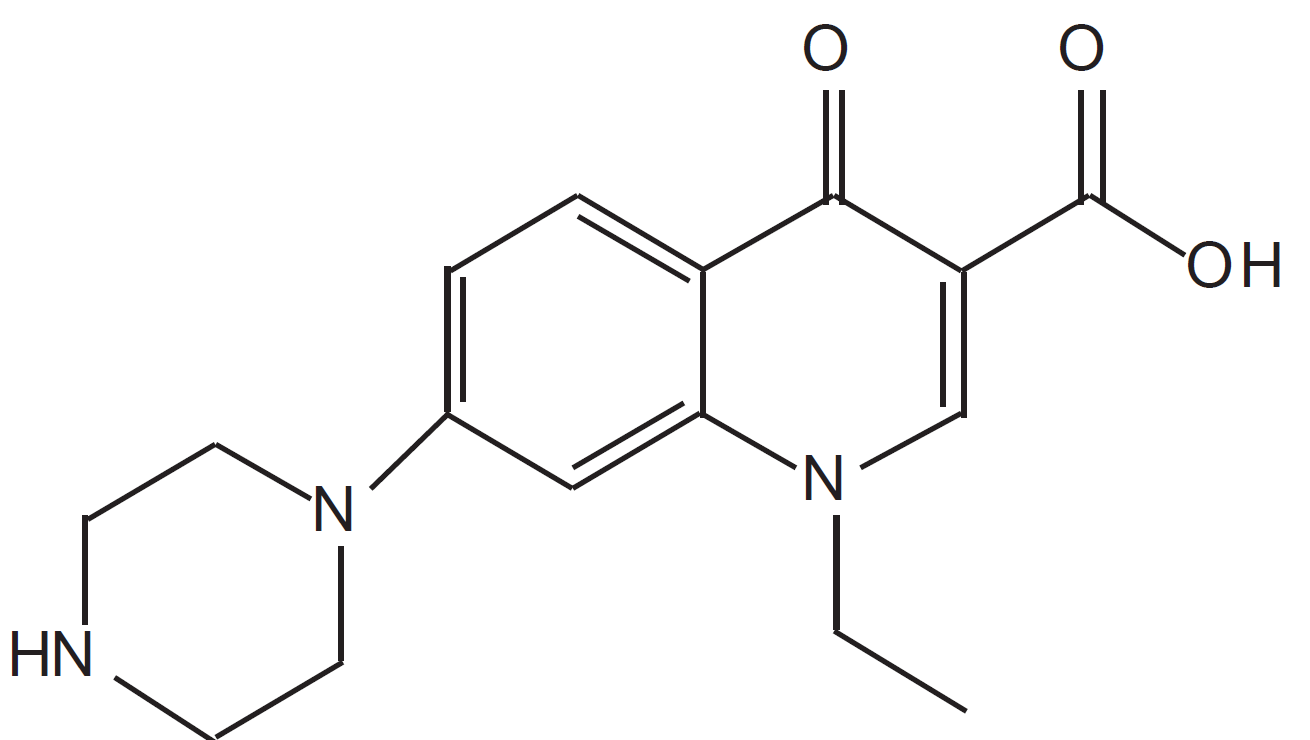 |
| N9 | 13.31 | C_14_H_14_FN_3_O_3_ | 292.22 | 291.22 | 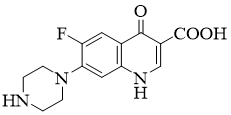 |
